# Non-Linear Association of Dietary Polyamines with the Risk of Incident Dementia: Results from Population-Based Cohort of the UK Biobank

**DOI:** 10.3390/nu16162774

**Published:** 2024-08-20

**Authors:** Mingxia Qian, Na Zhang, Rui Zhang, Min Liu, Yani Wu, Ying Lu, Furong Li, Liqiang Zheng

**Affiliations:** 1 School of Public Health, Shanghai Jiao Tong University School of Medicine, No. 280 South Chongqing Road, Huangpu District, Shanghai 200025, China; qianmx2000@126.com (M.Q.); nazhang2021@shsmu.edu.cn (N.Z.); soulnsn@sjtu.edu.cn (Y.W.); 2 College of Public Health, Shanghai University of Medicine and Health Sciences, No. 279 Zhouzhu Road, Pudong New District, Shanghai 201318, China; gina_zr@126.com; 3 Department of Epidemiology, School of Public Health, China Medical University, No. 77 Puhe Road, Shenyang 110122, China; 17725165216@163.com; 4 Department of Physical and Chemical, Changning District Center for Disease Control and Prevention, Shanghai 200051, China; yinzru@163.com; 5 School of Public Health and Emergency Management, Southern University of Science and Technology, No. 1088 Xueyuan Avenue, Nanshan District, Shenzhen 518055, China

**Keywords:** dementia, polyamines, spermidine, spermine, putrescine

## Abstract

Natural polyamines, including spermidine (SPD), spermine (SPM) and putrescine (PUT), are evolutionarily conserved endogenous molecules crucially involved in central cellular processes. Their physiological importance may extend to the maintenance of cognitive function during aging. However, limited population-based epidemiological studies have explored the link between dietary polyamines and dementia risk. This study was a prospective analysis of 77,092 UK Biobank participants aged ≥ 60 years without dementia at baseline. We used Cox proportional hazard regression models to explore the associations between dietary polyamines and the risk of dementia, and restricted cubic splines to test the non-linear relationships. During a median follow-up of 12 years, 1087 incidents of all-cause dementia cases occurred, including 450 Alzheimer’s disease (AD) cases and 206 vascular dementia (VD) cases. The fully adjusted hazard ratios (HRs) for the upper fourth quintile of dietary SPD, in comparison with the lowest quintile of intake, were 0.68 (95% confidence interval [95% CI]: 0.66–0.83) for the risk of all-cause dementia, 0.62 (95% CI: 0.45–0.85) for AD and 0.56 (95% CI: 0.36–0.88) for VD, respectively. A 26% reduction in dementia risk [HR: 0.74, (95% CI: 0.61–0.89)] and a 47% reduction in AD [HR: 0.53, (95%CI: 0.39–0.72)] were observed comparing the third with the lowest quintiles of dietary SPM. Dietary PUT was only associated with a reduced risk of all-cause dementia in the fourth quintile [HR (95% CI): 0.82 (0.68–0.99)]. Reduced risk was not found to be significant across all quintiles. There were ‘U’-shaped relationships found between dietary polyamines and all-cause dementia, AD and VD. Stratification by genetic predisposition showed no significant effect modification. Optimal intake of polyamines was linked to a decreased risk of dementia, with no modification by genetic risk. This potentially suggests cognitive benefits of dietary natural polyamines in humans.

## 1. Introduction

Dementia is characterized by the progressive deterioration of mental abilities, significantly impacting independent living [1]. The number of people aged 65 years and older is believed to be going to increase to 19.3% by 2030, and about 1% of people will suffer from dementia at the age of 60 [2]. Alzheimer’s disease (AD) and vascular dementia (VD) are the predominant dementia types [1], accounting for 60–70% [3] and 15–30% [4,5] of all-cause dementia, respectively. Compelling evidence supports the potential of distinct nutritional factors in maintaining cognitive function throughout aging [6]. The polyamines spermidine (SPD), spermine (SPM) and putrescine (PUT) are vital cationic molecules, involved in cell proliferation and differentiation, among many other cellular functions [2]. Although cellular and microbial synthesis in the gut are the main sources of polyamines, their biosynthesis alone is insufficient to maintain cell growth [7]. Hence, it is crucial to obtain polyamines through dietary intake.

The naturally occurring polyamines, especially SPD, play an essential role in various cellular processes, including autophagy and the maintenance of cellular homeostasis [8,9,10]. Animal studies have revealed that reduced polyamine levels were associated with declining memory function [10,11,12], whereas external supplementation of dietary polyamines would restore endogenous SPD levels in the brains of fruit flies, thereby preventing age-related memory impairment [11,12]. Recently, late-in-life SPD supplementation improved the cognitive function of aged wild-type mice via improved mitochondrial function [13,14], which is corroborated by epidemiological studies demonstrating the protective effects of SPD on mammalian cognition [15,16]. However, these studies mainly focused on exploring the relationship between blood SPD levels and cognitive function in relatively healthy human cohorts [16]. One study found reduced cognitive impairments in humans with higher dietary SPD intake [13]. Furthermore, randomized controlled trials (RCTs) support exogenous SPD supplementation for subject cognitive decline (SCD) [15,17] and brain health [18] in older adults. However, the impact of dietary SPD intake on dementia and its subtypes is yet to be determined. Additionally, despite animal studies revealing the neuroprotective effects of two other types of polyamines, SPM and PUT [19,20,21], little research has documented the relationship between their dietary intake with dementia risk.

Here, we examined the association between the intake of dietary SPD, SPM and PUT and the risk of dementia in a large population-based study. Moreover, we also investigated whether genetic predisposition has a modification effect among them.

## 2. Materials and Methods

This large-scale cohort study utilized data from the UK Biobank (Application Number 60009), which obtained approval from the National Information Governance Board for Health and Social Care and the National Health Service North West Multicenter Research Ethics Committee (11/NW/0382). All participants provided informed consent through electronic signature at baseline assessment. A comprehensive list of available resources can be found on the UK Biobank website. (https://www.ukbiobank.ac.uk/, accessed on 1 January 2021).

### 2.1. Study Population

The UK Biobank is a population-based cohort comprising over 500,000 participants aged 37–73 years, recruited from 22 assessment centers across the United Kingdom between 2006 and 2010 [22,23]. Our analyses were restricted to individuals aged at least 60 years at baseline (n = 285,012), as the majority of incident dementia cases occur in older adults. Participants were excluded from the analyses if they had missing dietary data (n = 122,406), exhibited an abnormal energy range [24] (defined as <3349 KJ/day or >20,934 KJ/day for males and <2093 KJ/day or >16,747 KJ/day for females) (n = 3387), had dementia at baseline (n = 19), lacked APOE *ε*4 and the polygenic risk score (PRS) information (n = 5731) or displayed extreme values (beyond ±4 standard deviations (SD) from the mean) in dietary SPD, SPM and PUT data (n = 1320). The final analyses included 77,029 individuals (Figure S1).

### 2.2. Exposure Assessment

Dietary intake data were evaluated through a web-based, self-administered questionnaire aimed at recording the consumption of more than 200 typical food and beverage items over the past 24 h. This 24 h dietary recall questionnaire was first introduced toward the end of the recruitment period (2009–2010). Subsequently, all participants with a registered email address were invited to fill out the online questionnaire on four separate occasions between 2011 and 2012. Validated against an interviewer-administered 24 h recall [25], this web-based questionnaire demonstrated comparable results in capturing food and drink items, as well as estimating energy and nutrient intakes. Each participant reported information on portion size of dietary intake, and a nutrient database for dietary SPD, SPM and PUT was derived from published data in previous publications [26,27,28,29,30,31,32,33,34,35,36,37,38,39]. Total dietary SPD, SPM and PUT intake were from foods of plant origin, including vegetables, fruits, cereals, starchy food and nuts, and foods of animal origin, including meat, fish, eggs, yogurt/ice cream, cheese and other daily diets, including snacks, dessert, drinks and alcohol (Table S1). This information was utilized to calculate the average daily intake of each food item in nmol/day. Dietary SPD, SPM and PUT were categorized into quintiles (Q1–Q5), ranging from the lowest (Q1) to the highest (Q5) values.

### 2.3. Dementia Diagnosis

The primary outcome in this study was incident all-cause dementia, with secondary outcomes including AD and VD. All-cause dementia cases were ascertained through data linkage to hospital inpatient records, which included admission and diagnosis information from the Hospital Episode Statistics for England, Scottish Morbidity Record data for Scotland, and the Patient Episode Database for Wales. Additional cases were discovered through linkage to death register data provided by the National Health Service Digital for England and Wales and the Information and Statistics Division for Scotland. Participants with dementia were identified based on primary/secondary diagnosis (hospital records) or underlying/contributory cause of death (death register) using ICD-9 and ICD-10 codes for AD and VD (Table S2).

### 2.4. Polygenic Risk Score

The PRS was computed utilizing the same methods established in a prior study [40]. The PRS consists of 249,273 single-nucleotide polymorphisms (SNPs) associated with AD at *p* < 0.05 [41]. Each SNP was weighted by its effect on the risk of AD. Specifically, the PRS was calculated using the weighted formula by PLINK as follows: PRS = (β_1_ × SNP_1_ + β_2_ × SNP_2_ + … + β_249,273_ × SNP_249,273_)/2 × 249,273. The PRS was divided into quintiles and then categorized into low- (quintile 1), medium- (quintiles 2–4) and high-risk (quintile 5) groups.

### 2.5. APOE Genotyping

The three main APOE haplotypes (*ε*4, *ε*3, and *ε*2) are defined by combinations of two SNPs, rs429358 and rs7412, which were genotyped directly in the UKB. We excluded *ε*1*ε*4 and *ε*1*ε*2 genotypes from the subsequent analyses due to their small sample size. Moreover, *ε*2*ε*4/*ε*1*ε*3 genotypes were coded as *ε*2*ε*4 since the *ε*1 allele is rare. The final six APOE genotypes (*ε*4*ε*4, *ε*4*ε*3, *ε*4*ε*2, *ε*3*ε*3, *ε*2*ε*3 and *ε*2*ε*2) were grouped into three categories: zero, one and two *ε*4 alleles carriers.

### 2.6. Covariates

In the present study, we considered the following potential covariates: age, sex, socioeconomic status (categorized from quintiles 1, 2 to 4, and 5 of the Townsend deprivation index [42], combining information on social class; employment; car availability; housing; education (categorized as higher (college/university degree), other (A levels/AS levels or equivalent, O levels/GCSEs or equivalent, CSEs or equivalent, NVQ or HND or HNC or equivalent, other professional qualifications)); smoking status (never/previous/current); alcohol intake (<1 time/week, 1–2 times/week, 3–4 times/week, daily or almost daily); energy; sleep duration (<7 h/day, 7–8 h/day and >8 h hours/day); physical activity levels (low: <1200 (metabolic equivalent task (MET) minutes per week for all activity), high: ≥1200 MET min/week, recorded the frequency, duration and intensity of walking, moderate and vigorous activity); hypertension (no/yes); hypercholesteremia (no/yes); diabetes (no/yes) and the first 10 principal components of ancestry. All measures were obtained using the baseline questionnaire.

### 2.7. Statistical Analysis

Baseline characteristics of the analytic sample were summarized across quintiles of dietary SPD, SPM and PUT. Categorical variables were presented as percentages, while normally distributed continuous variables were expressed as mean and SD. Group differences across quintiles were assessed using analysis of variance or χ^2^ tests, as appropriate.

The restricted cubic spline model (RCS) evaluated the relationship between dietary SPD, SPM and PUT and the risk of all-cause dementia, AD and VD. Knots were placed at the 25th, 50th, 75th, and 95th percentiles of the exposure distribution. Cox proportional hazard regression models were used to estimate the relationship between dietary SPD, SPM and PUT and the risk of all-cause dementia, AD and VD. Hazard ratios (HRs) and corresponding 95% confidence intervals (CIs) were calculated in the present analysis. Two models were employed: the minimally adjusted model (model 1) was adjusted for age and sex only. The fully adjusted model (model 2) was adjusted for age, sex, socioeconomic status, education, smoking status, alcohol intake, energy, sleep duration, physical activity levels, polygenic risk score, number of APOE *ε*4, hypertension, hypercholesteremia, diabetes and the first 10 principal components of ancestry.

Missing information was addressed by the median values for continuous covariates and by using a missing indicator for categorical covariates (all covariates < 5% missing). Additional analyses were conducted to assess the robustness of the results. Stratified analyses tested the potential modification effects by factors including age, sex, socioeconomic status, education, alcohol intake, energy, sleep duration, physical activity level, polygenic risk score, APOE *ε*4, hypertension, hypercholesteremia and diabetes. Multiplicative interactions were examined by adding a multiplicative term in the Cox regression models. Several sensitivity analyses were performed as follows: (1) restricting participants to those with a follow-up period of ≥2 and ≥5 years, respectively; (2) excluding participants with one follow-up; (3) excluding participants in the top and bottom 5% and 10% of dietary SPD, SPM and PUT, respectively. *p* values were 2-sided, with statistical significance set at less than 0.05. All analyses were performed using R 4.2.2 version.

## 3. Results

### 3.1. Cohort Characteristics

A total of 77,092 individuals were included in our final analyses (Figure S1). Baseline characteristics of the participants were stratified by quintiles of dietary SPD, SPM and PUT intake (Table 1). Among the participants, the mean age was 63.9 ± 2.8 years, and 39,427 (51.1%) were female (Table 1). Significant, but subtle, differences existed in age, sex, socioeconomic status, education, smoking status, alcohol intake, energy, physical activity level, sleep duration, hypertension, hypercholesterolemia, diabetes and BMI among SPD, SPM and PUT quintiles, respectively (all *p* < 0.05) (Table 1).

During a mean follow-up of 12 years, 1087 cases of all-cause dementia were identified, including 450 AD and 206 VD cases (Table S3). The incidence rate of all-cause dementia (cases per 1000 person-years) was 1.39, 1.05, 1.08, 0.95 and 1.33 for the first, second, third, fourth and fifth quintile of dietary SPD intake, respectively (Table S3). For SPM intake, the incidence rate of all-cause dementia (cases per 1000 person-years) was 1.36, 1.02, 1.02, 1.15 and 1.23 for the first, second, third, fourth and fifth quintiles, respectively (Table S3). Additionally, for the PUT intake, the incidence rate of all-cause dementia (cases per 1000 person-years) was 1.34, 1.02, 1.16, 1.09 and 1.17 for the first, second, third, fourth and fifth quintiles, respectively (Table S3).

### 3.2. Association of Dietary SPD with Incidence of All-Cause Dementia, AD and VD

**Figure 1:**
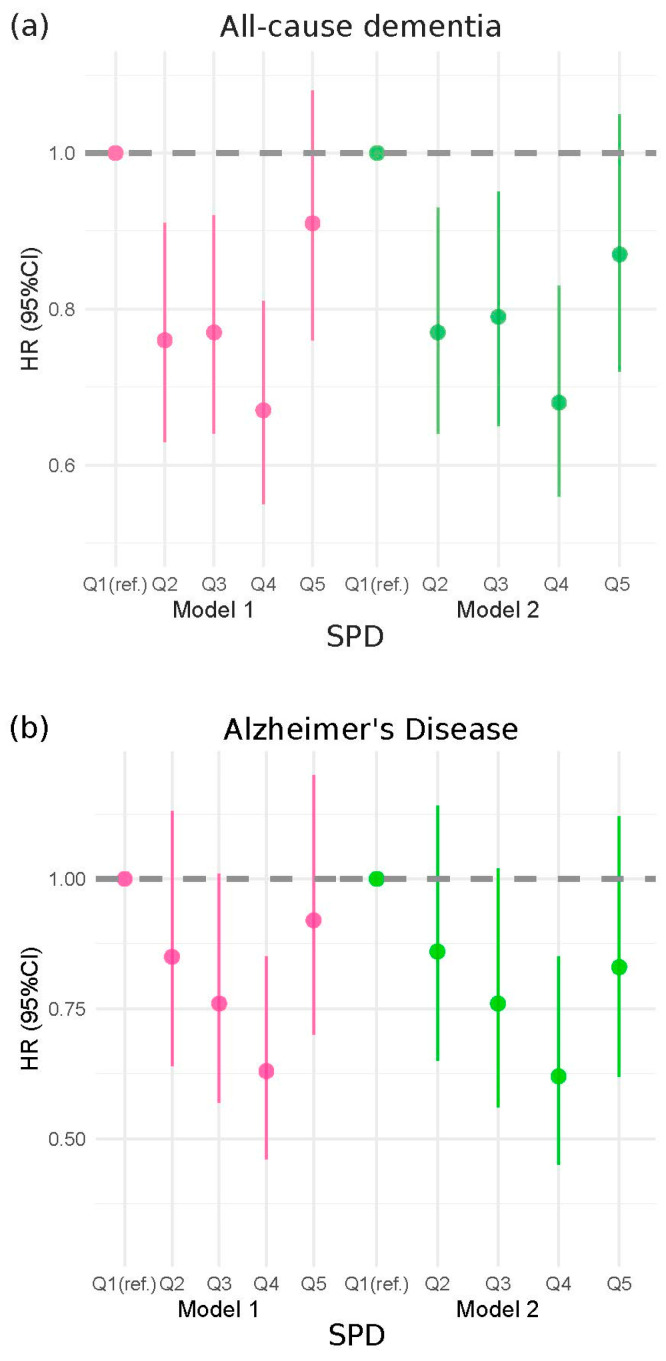

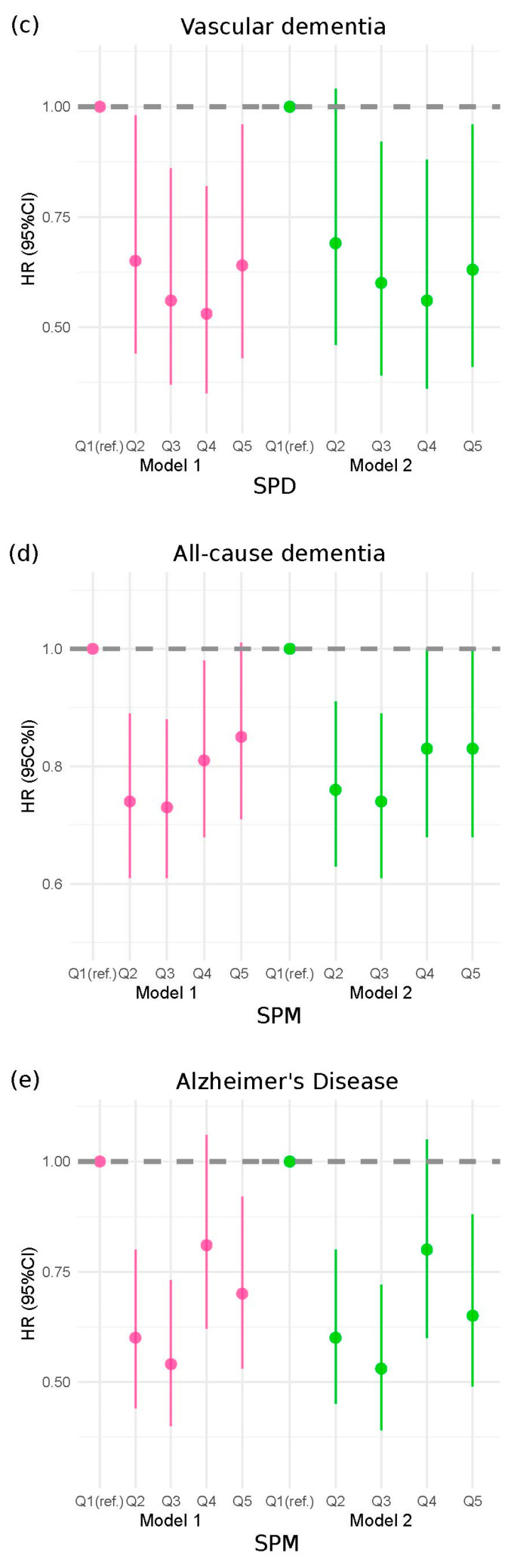

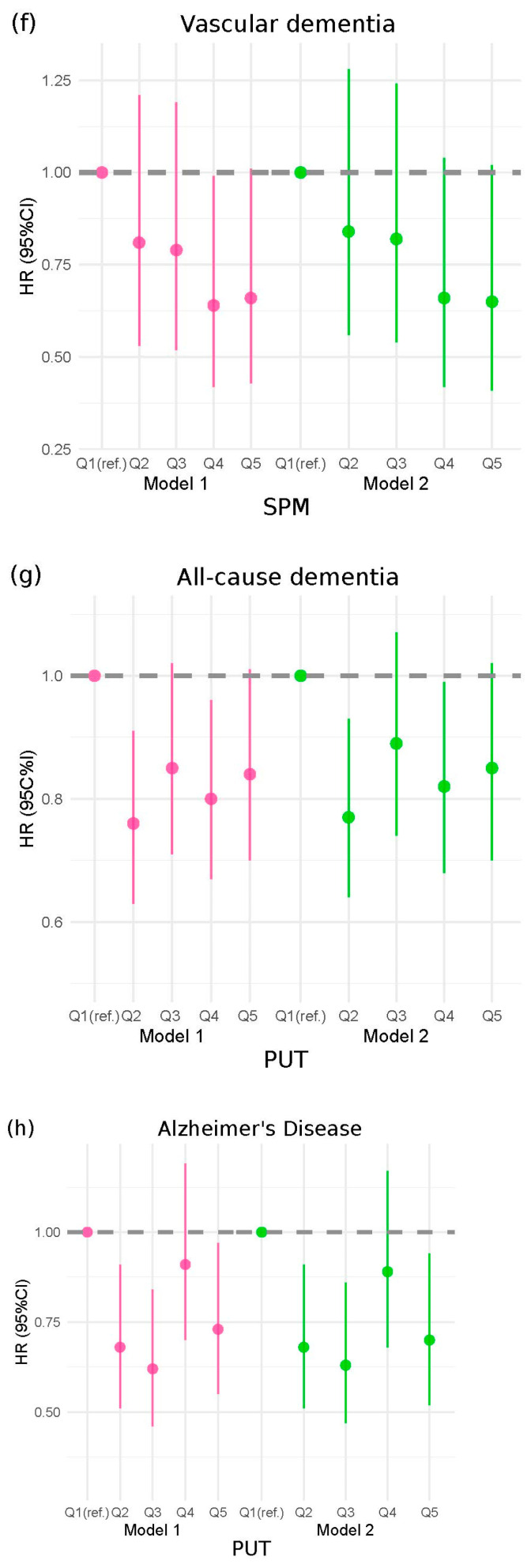

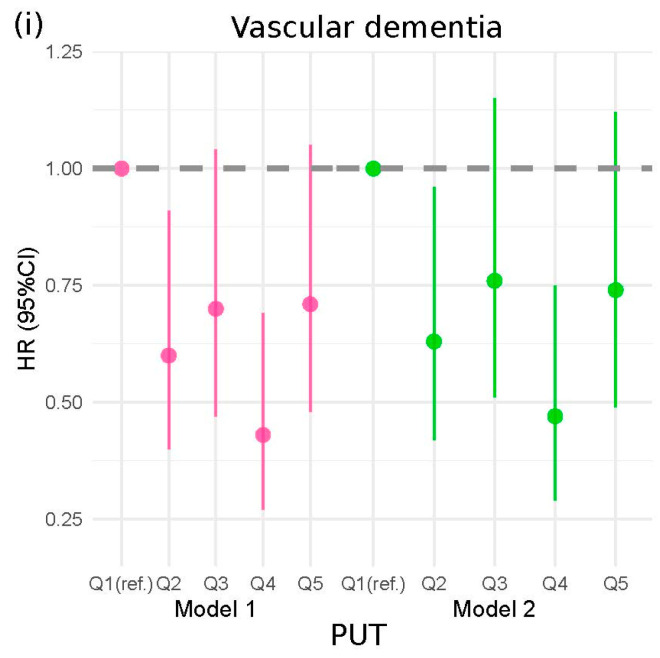
Cox proportional hazards models investigating the association between dietary spermidine, spermine and putrescine intake and risk of all-cause dementia and cause-specific dementia (Alzheimer’s disease (AD) and vascular disease (VD)): (**a**) spermidine and all-cause dementia; (**b**) spermidine and AD; (**c**) spermidine and VD; (**d**) spermine and all-cause dementia; (**e**) spermine and AD; (**f**) spermine and VD; (**g**) putrescine and dementia; (**h**) putrescine and AD; (**i**) putrescine and VD. HR: hazard ratio; CI, confidence interval. Model 1: Cox proportional hazards regression adjusted for age, sex; Model 2: Cox proportional hazards regression adjusted for Model 1, socioeconomic status (least deprived/medium/most deprived), education (high/other), smoking status (never/previous/current), alcohol intake (<1 time/week, 1–2 times/week, 3–4 times/week, daily or almost daily), energy, sleep duration(<7 h/7–8 h/>8 h), physical activity levels (moderate/low/high), polygenic risk score, APOE *ε*4, hypertension (no/yes), hypercholesteremia (no/yes), diabetes (no/yes) and first 10 principal components of ancestry.

**Figure 2:**
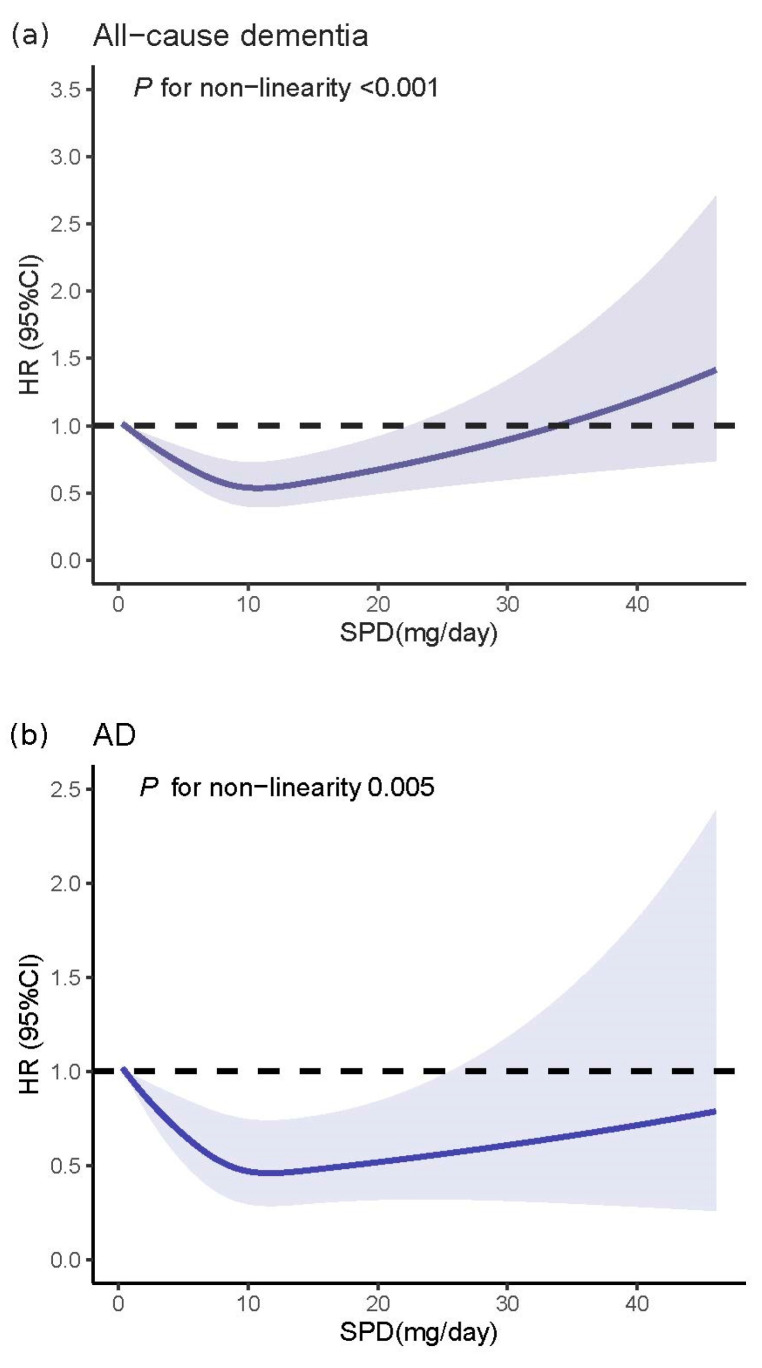

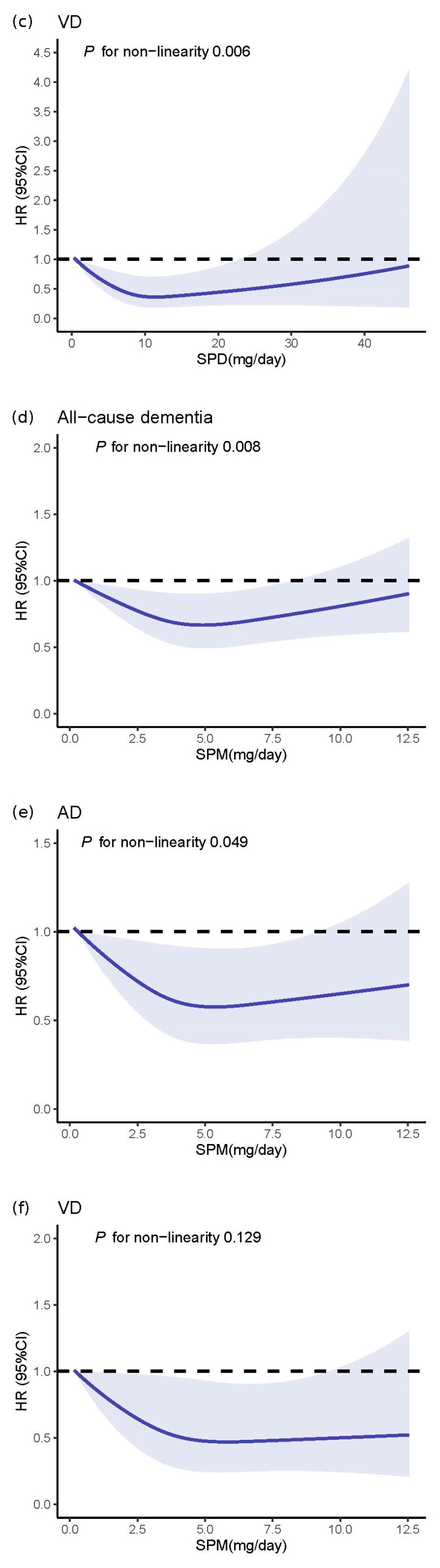

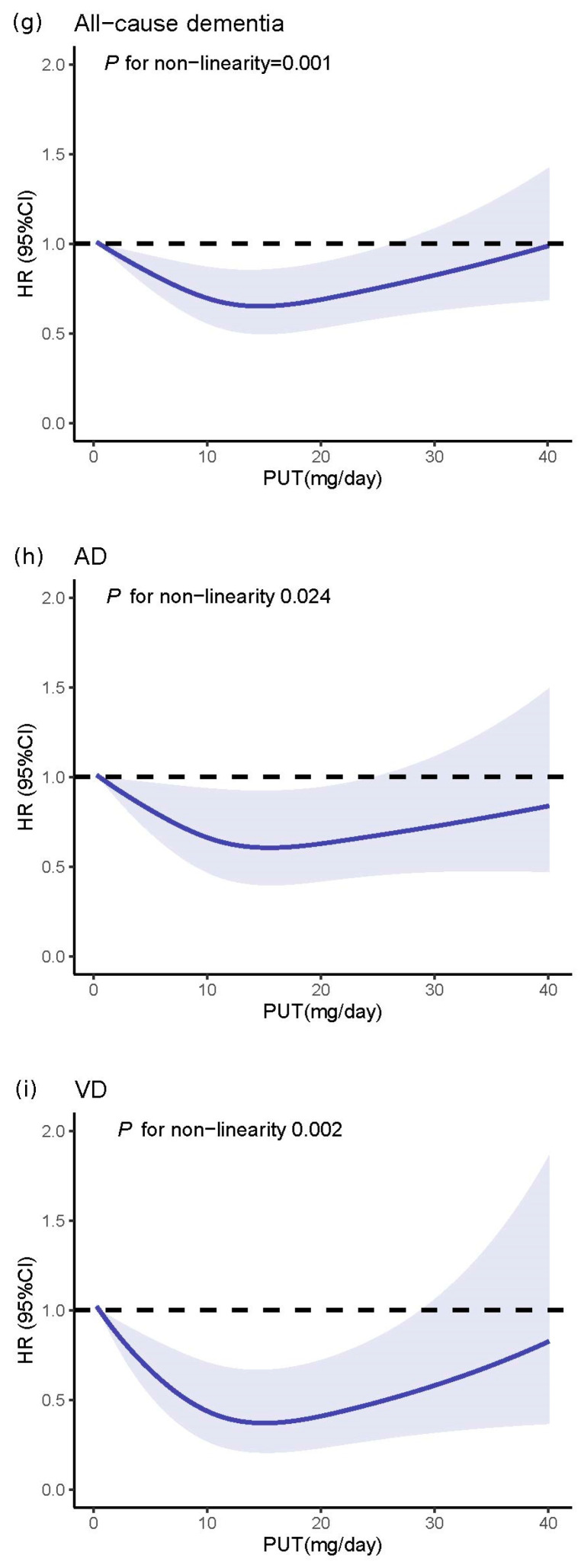
Restricted cubic spline models for the relationship between dietary spermidine, spermine and putrescine intake and risk of all-cause dementia and cause-specific dementia (Alzheimer’s disease (AD) and vascular disease (VD)): (**a**) spermidine and all-cause dementia; (**b**) spermidine and AD; (**c**) spermidine and VD; (**d**) spermine and all-cause dementia; (**e**) spermine and AD; (**f**) spermine and VD; (**g**) putrescine and dementia; (**h**) putrescine and AD; (**i**) putrescine and VD. The 95% CIs of the adjusted HRs are represented by the shaded area. HR, hazard ratio; CI, confidence interval. The model is adjusted by age, sex, socioeconomic status (least deprived/medium/most deprived), education (high/other), smoking status (never/previous/current), alcohol intake (<1 time/week, 1–2 times/week, 3–4 times/week, daily or almost daily), energy, sleep duration (<7 h/7–8 h/>8 h), physical activity levels (moderate/low/high), polygenic risk score, APOE *ε*4, hypertension (no/yes), hypercholesteremia (no/yes), diabetes (no/yes) and first 10 principal components of ancestry.

Compared with the first quintile of dietary SPD intake (≤6.5 mg/day), full-adjusted HRs for incident dementia were lower in the fourth quintile (10.0–12.3 mg/day): 0.68 (95% CI: 0.66–0.83) for the risk of all-cause dementia, 0.62 (95% CI: 0.45–0.85) for the risk of AD and 0.56 (95% CI: 0.36–0.88) for the risk of VD, respectively. However, the highest intake of SPD (>12.3 mg/day) had no protective role on the risk of all-cause dementia [0.87 (95% CI: 0.72–1.05)] and on the risk of VD [0.83 (95%CI: 0.62–1.12)], respectively (Figure 1). The multivariable-adjusted restricted cubic spline model indicated significant non-linear associations between dietary SPD and the risk of all-cause dementia (*p* for non-linearity < 0.001), AD (*p* for non-linearity = 0.008) and VD (*p* for non-linearity = 0.001) (Figure 2).

### 3.3. Association of Dietary SPM with Incidence of All-Cause Dementia, AD and VD

Dietary SPM was inversely related to all-cause dementia and AD (Figure 1). When compared to the first quintile group (≤2.7 mg/day) of dietary SPM intake, the fourth quintile group (4.4–5.7 mg/day) had a 26% reduced risk of all-cause dementia [0.74 (95%CI: 0.61–0.89)] and a 47% reduced risk of AD [0.53 (95%CI: 0.39–0.72)]. There was no statistical significance for the risk of VD [0.82 (0.54–1.24)] in the fully adjusted model. Moreover, no significant differences were detected in the risk of all-cause dementia in the fifth quintile group (≥5.7 mg/day) compared to the first quintile group, either in the simple [0.85 (95%CI: 0.71–1.01] or fully adjusted model [0.83 (95%CI: 0.68–1.00)] (Figure 1). The RCS models indicated significant non-linear associations between dietary SPM and all-cause dementia (*p* for non-linearity = 0.005; Figure 2), AD (*p* for non-linearity = 0.049; Figure 2) and VD (*p* for non-linearity = 0.024; Figure 2).

### 3.4. Association of Dietary PUT with Incidence of All-Cause Dementia, AD and VD

Participants in the fourth quintile group (13.4 mg/day–17.8 mg/day) of dietary PUT intake had a lower hazard of all-cause dementia compared with the lowest quintile group (≤7.6 mg/day), with an HR of 0.80 (95%CI: 0.67–0.96) and 0.82 (95%CI: 0.68–0.99) in the simple and full-adjusted model, respectively. No significant associations were found in other quintile groups (all *p* > 0.05) (Figure 1). No significant correlation was found between dietary PUT and the risk of AD in either the simple or full-adjusted model (both *p* > 0.05) (Figure 1). Participants in the fourth quintile group had a lower risk of VD compared with the lowest quintile group in the simple model [0.43 (95%CI: 0.27–0.69)], whereas no significant associations were found in other quintile groups (all *p* > 0.05). Similar findings were found in the full-adjusted model (*p* > 0.05) (Figure 1). Dietary PUT exhibited a non-linear association with the risk of all-cause dementia (*p* for non-linearity = 0.006) and VD (*p* for non-linearity = 0.001), which was not observed with AD (*p* for non-linearity = 0.123) (Figure 2).

### 3.5. Subgroup and Sensitivity Analyses

**Figure 3:**
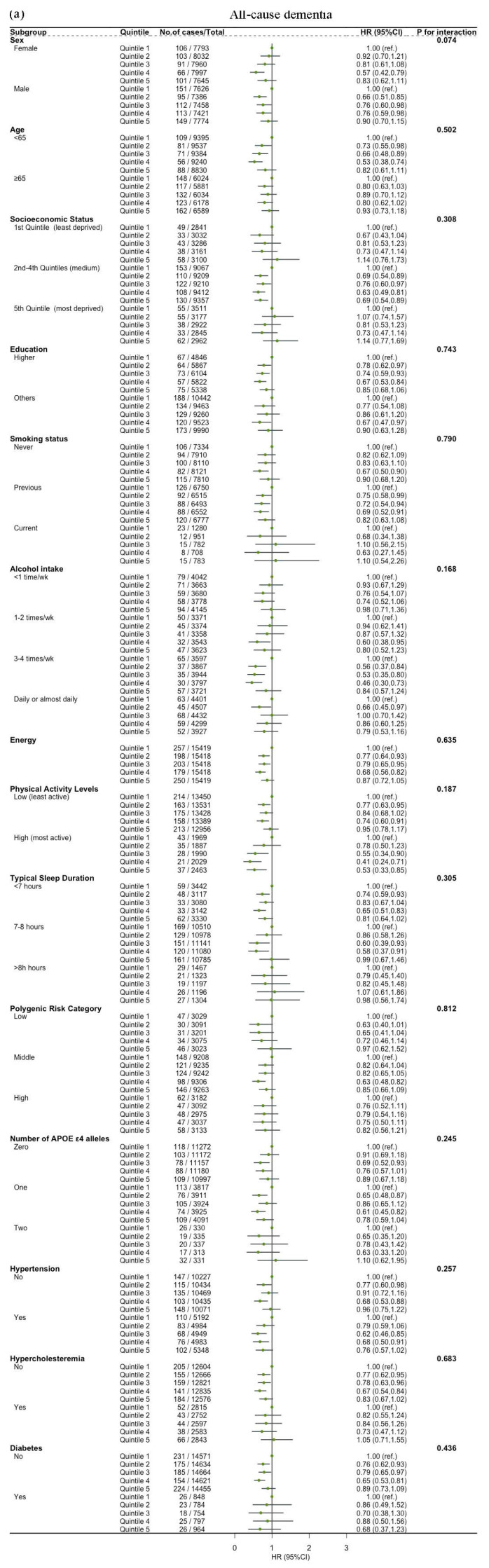

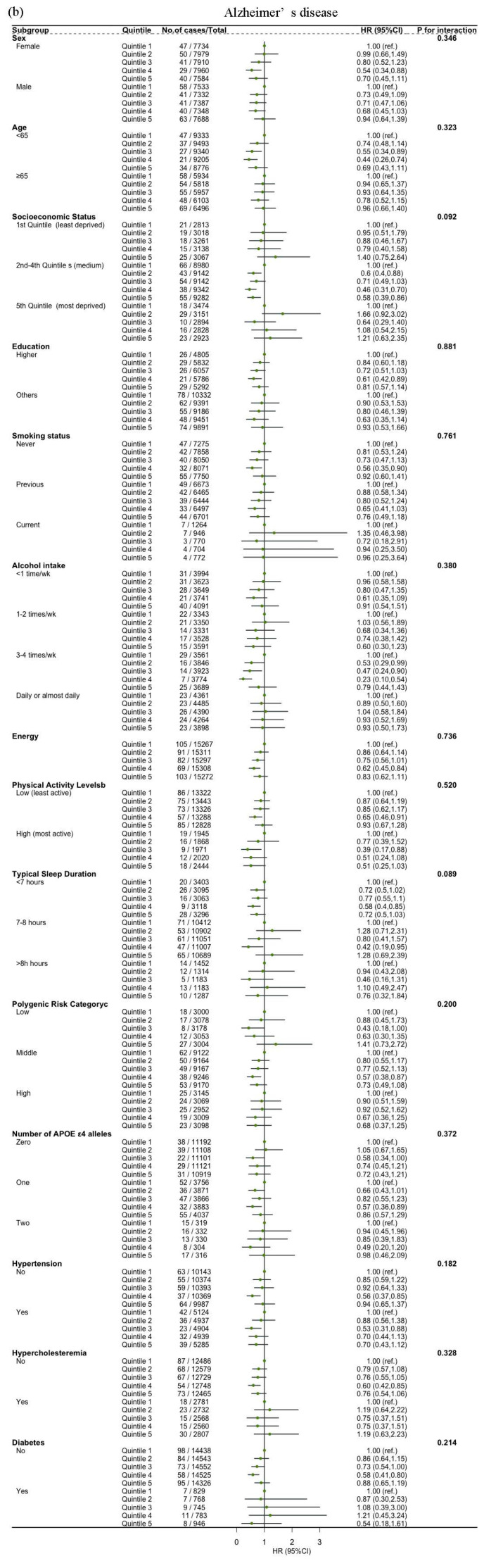

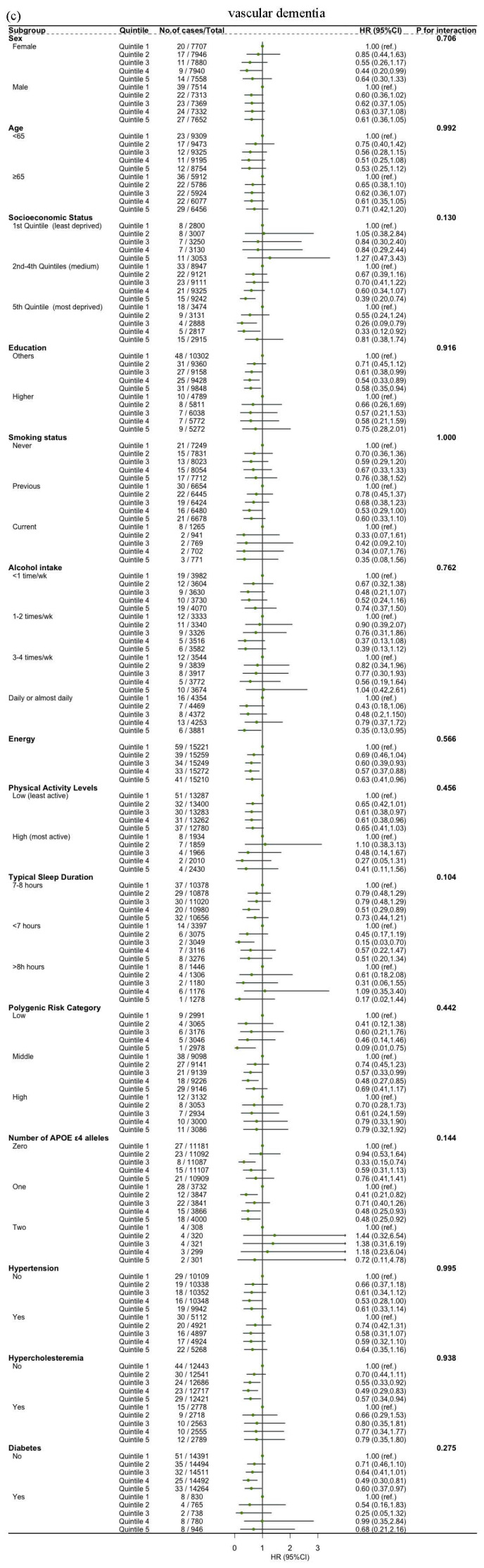
Association of dietary spermidine with all-cause dementia and cause-specific dementia, stratified by potential risk factors: (**a**) spermidine and all-cause dementia; (**b**) spermidine and AD; (**c**) spermidine and VD. HR, hazard ratio; CI, confidence interval. The model is adjusted by age, sex, socioeconomic status (least deprived/medium/most deprived), education (high/other), smoking status (never/previous/current), alcohol intake (<1 time/week, 1–2 times/week, 3–4 times/week, daily or almost daily), energy, sleep duration (<7 h/7–8 h/>8 h), physical activity levels (moderate/low/high), polygenic risk score, APOE *ε*4, hypertension (no/yes), hypercholesteremia (no/yes), diabetes (no/yes) and first 10 principal components of ancestry.

**Figure 4:**
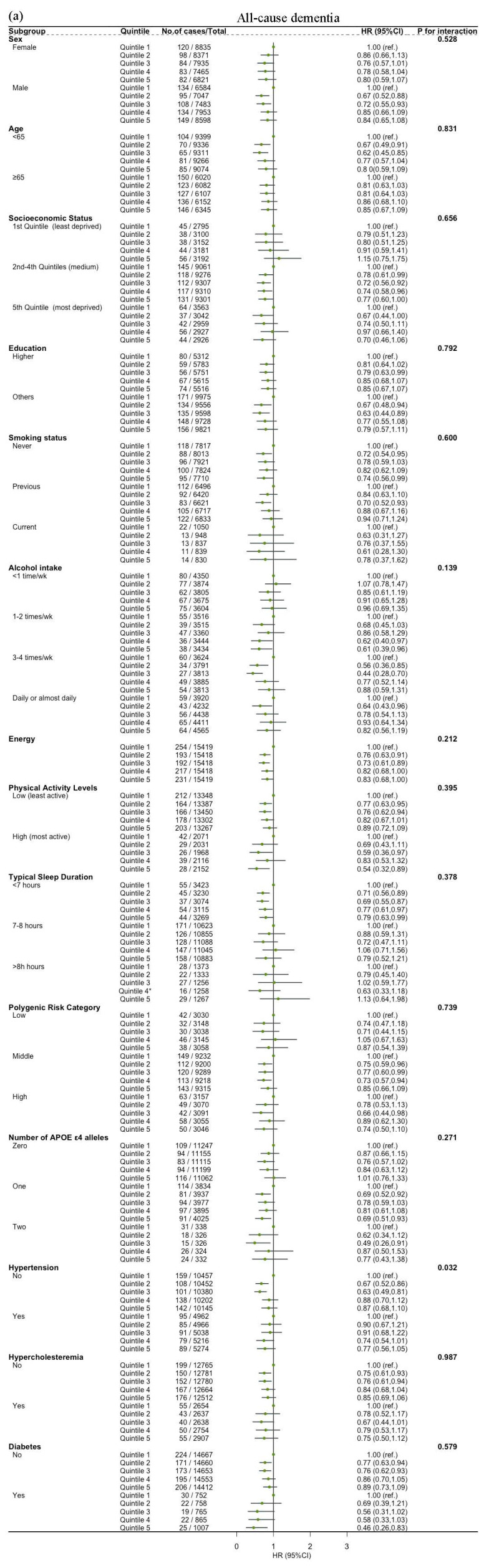

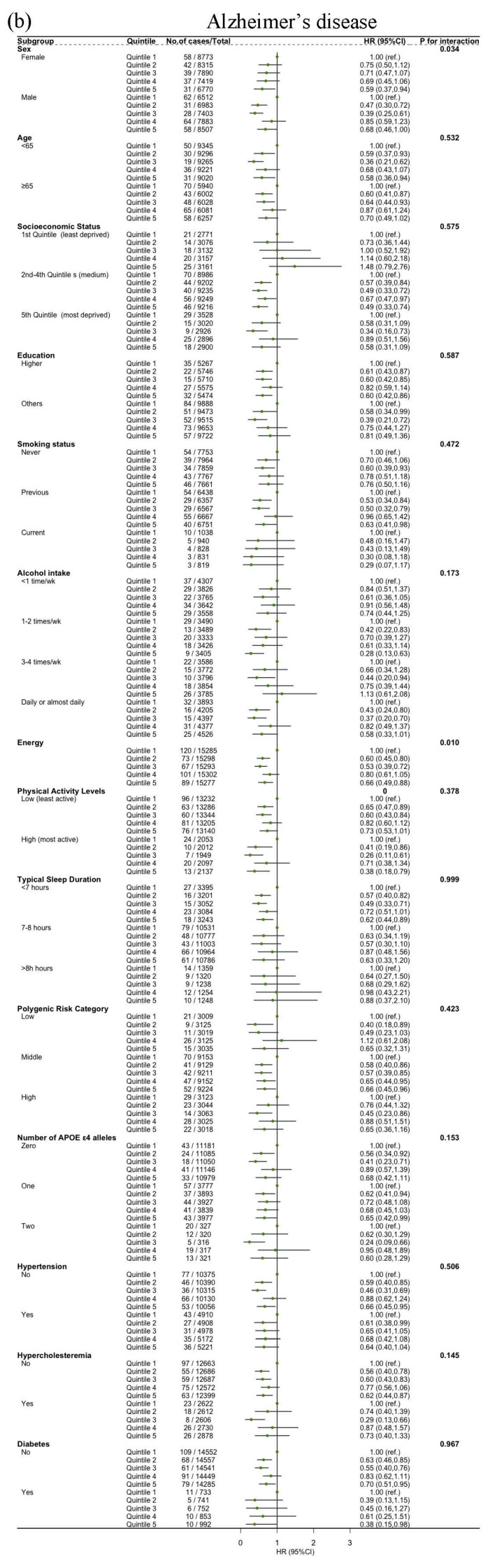

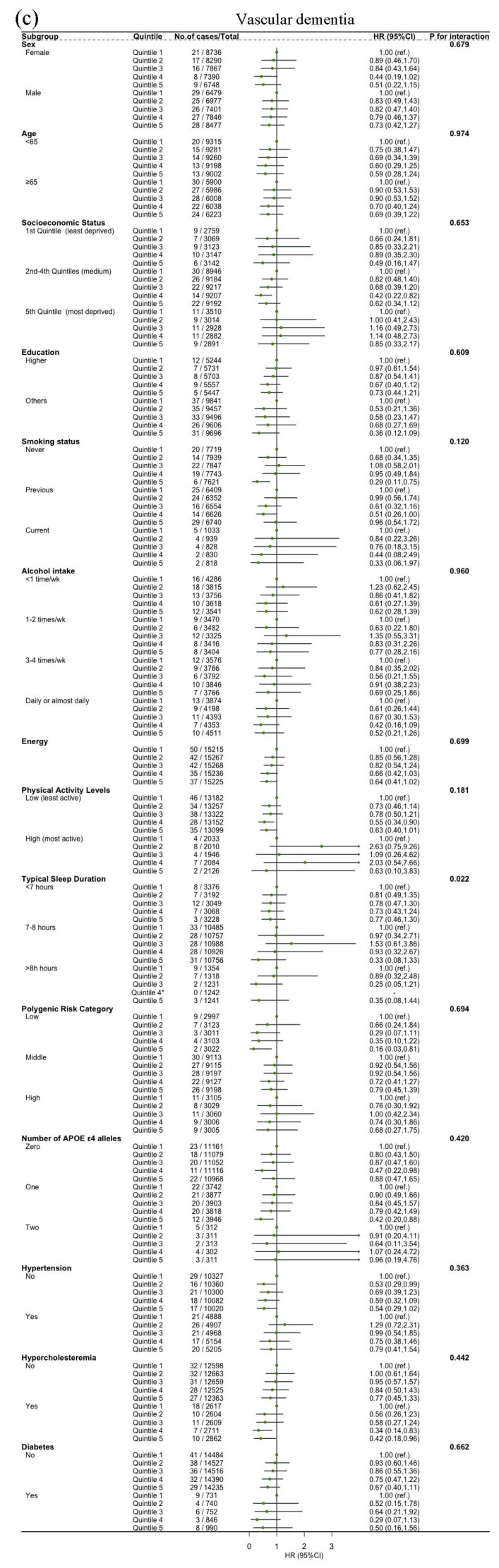
Association of dietary spermine with all-cause dementia and cause-specific dementia, stratified by potential risk factors: (**a**) spermine and all-cause dementia; (**b**) spermine and AD; (**c**) spermine and VD. HR, hazard ratio; CI, confidence interval. The model is adjusted by age, sex, socioeconomic status (least deprived/medium/most deprived), education (high/other), smoke status (never/previous/current), alcohol intake (<1 time/week, 1–2 times/week, 3–4 times/week, daily or almost daily), energy, sleep duration (<7 h/7–8 h/>8h), physical activity levels (moderate/low/high), polygenic risk score, APOE *ε*4, hypertension (no/yes), hypercholesteremia (no/yes), diabetes (no/yes) and first 10 principal components of ancestry.

**Figure 5:**
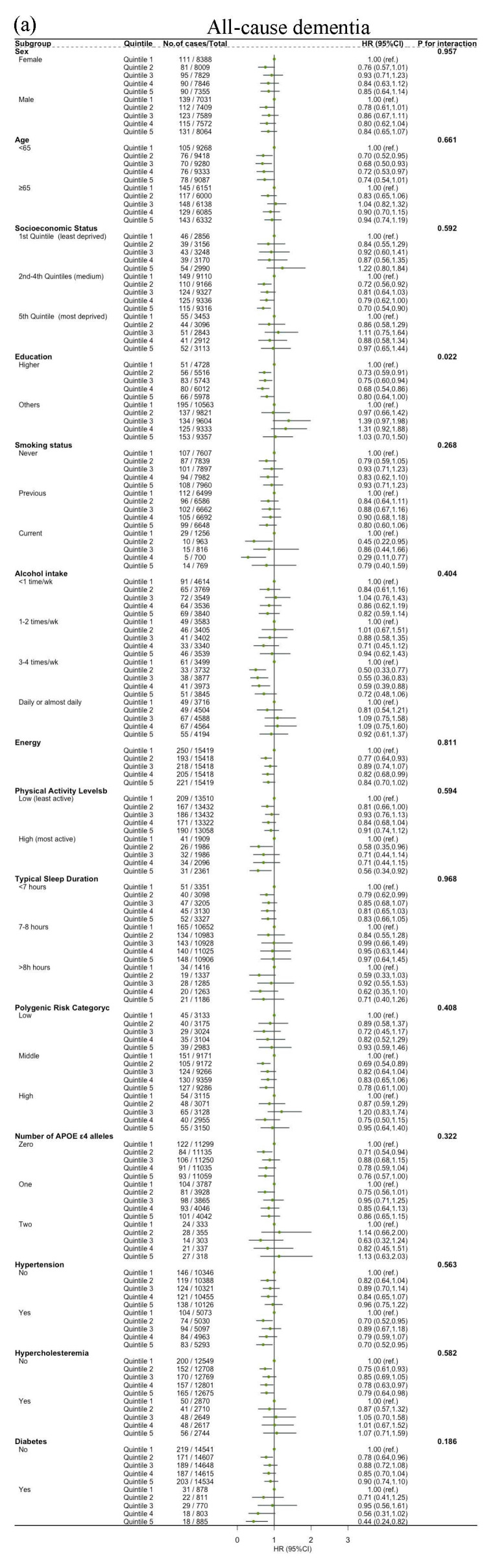

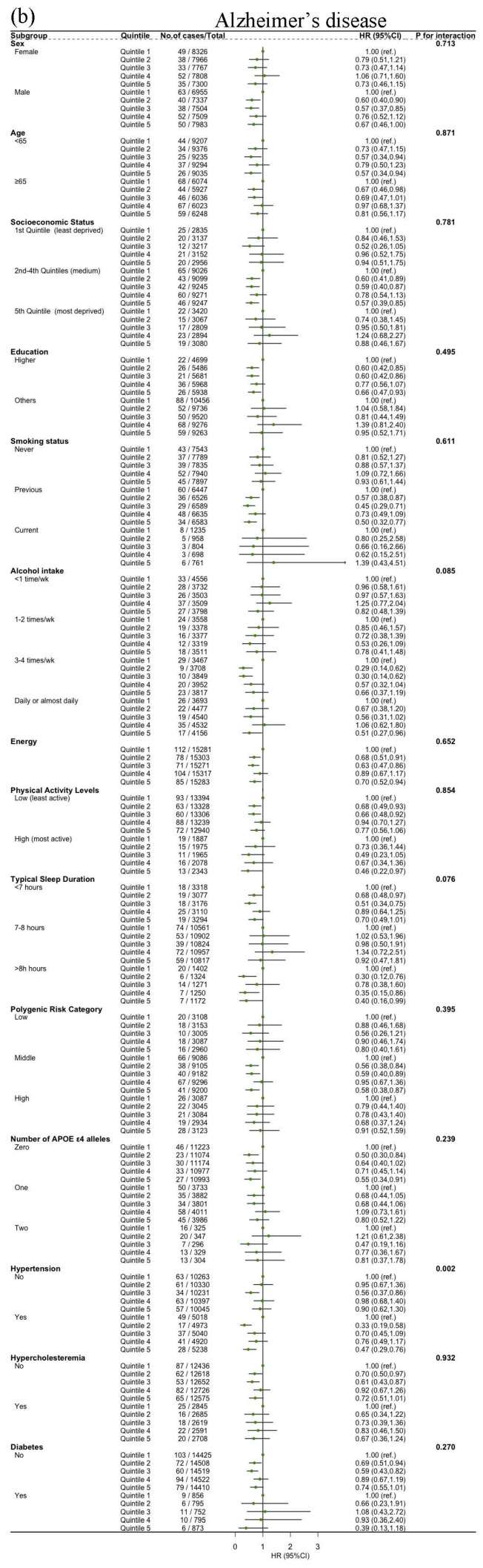

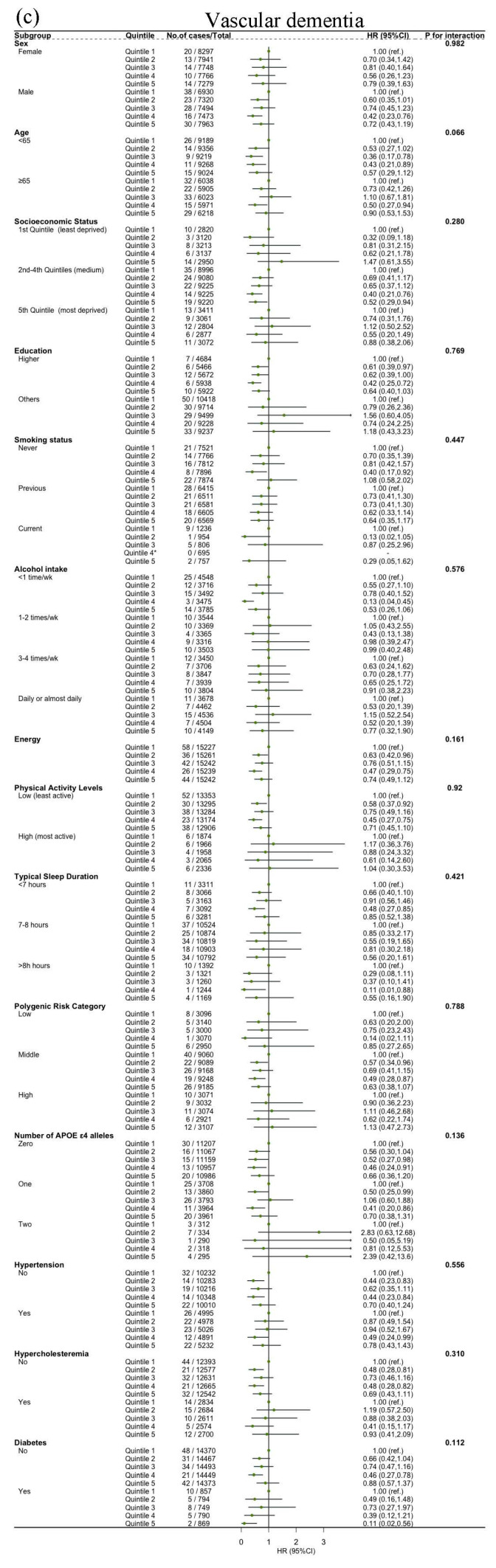
Association of dietary putrescine with all-cause dementia and cause-specific dementia stratified by potential risk factors: (**a**) putrescine and all-cause dementia; (**b**) putrescine and AD; (**c**) putrescine and VD. HR, hazard ratio; CI, confidence interval. The model is adjusted by age, sex, socioeconomic status (least deprived/medium/most deprived), education (high/other), smoking status (never/previous/current), alcohol intake (<1 time/week, 1–2 times/week, 3–4 times/week, daily or almost daily), energy, sleep duration (<7 h/7–8 h/>8 h), physical activity levels (moderate/low/high), polygenic risk score, APOE *ε4*, hypertension (no/yes), hypercholesteremia (no/yes), diabetes (no/yes) and first 10 principal components of ancestry.

The associations between dietary SPD intake and the risk of all-cause dementia, AD and VD were not modified by age, sex, socioeconomic status, education, alcohol intake, energy, sleep duration, physical activity level, polygenic risk score, number of APOE *ε*4, hypertension, hypercholesteremia and diabetes (all *p* for interaction > 0.05) (Figure 3). Significant interactions were observed between dietary SPM intake and hypertension, sex and energy on the risk of all-cause dementia (*p* for interaction = 0.032, 0.034, 0.001, respectively). Sleep duration influenced the relationship between dietary SPM intake and risk of VD (*p* = 0.022 for interaction) (Figure 4). Education affected the link between dietary PUT intake and risk of all-cause dementia (*p* = 0.022 for interaction). Hypertension modified the relationship between dietary PUT intake and risk of AD (*p* = 0.002 for interaction). Stratification by genetic predisposition showed no significant effect modification (all *p* for interaction > 0.05) (Figure 5).

In our sensitivity analyses, the associations between dietary SPD, SPM and PUT and dementia outcomes remained consistent: first, when restricting the follow-up time to ≥2 or 5 years, HRs remained similar to the main results in fully adjusted models (Tables S4 and S5); second, after excluding dementia cases that occurred within the first follow-up year, HRs were similar to those of the main results (Table S6); and third, removing participants within the top and bottom 10% of dietary SPD, SPM and PUT did not diminish the statistical significance for all-cause dementia, AD or VD (Tables S7 and S8). Additionally, adjusting for BMI, stroke and depression did not change the associations significantly (Tables S9–S11).

## 4. Discussion

This is the first population-based study to investigate the association between dietary polyamines (SPD, SPM and PUT) intake and all-cause and cause-specific dementia among older adults. We found that the optimal intake of dietary SPD, SPM and PUT was associated with a decreased risk of all-cause dementia, AD and VD, and the associations were not modified by genetic predisposition.

Our findings are in line with previous studies that demonstrate the protective effects of SPD supplementation on cognitive function in older adults [17,43]. An RCT involving thirty participants [17] showed that nutritional SPD had a positive impact on memory performance in older adults with SCD, a population vulnerable to non-normative cognitive decline and eventual progression to AD [44], even in the absence of detectable objective deterioration in neuropsychological tests. Exogenous SPD intake may enhance or maintain memory, thereby reducing overall dementia risk. Furthermore, another RCT found that a daily 3.3 mg dosage of oral SPD supplementation enhanced cognitive performance in older adults with mild to moderate dementia at 3-month follow-up [43], with sustained neuroprotective effects observed at 12 months [45]. However, a 12-month RCT concluded that long-term SPD supplementation in participants with SCD did not modify memory performance when compared to the placebo group [15], while exploratory analyses indicated possible positive effects on inflammation and verbal memory [15]. The conflicting findings may be attributed to the insufficient daily dose of 0.9 mg of SPD in the latter study, which failed to yield substantial positive effects on memory function [46]. Here, we observed significantly lower risks of dementia from the first quintile (≤6.5 mg/day) of dietary SPD intake to the fourth quintile (10.0–12.3 mg/day), which corresponds to a larger difference in exogenous SPD supply than studied in most clinical trials.

Several molecular mechanisms may explain the inverse associations between dietary SPD intake and dementia outcomes. First, experimental evidence demonstrates that SPD plays a crucial role in promoting autophagy, exerting anti-inflammatory effects, and mitigating antioxidant-induced stress [8,19,47], all of which are associated with enhancing memory and brain function [8,11,19,47,48,49]. In addition, previous research has shown that SPD can extend the lifespan of different species, from yeast to rodents, and promote the emergence of age-related diseases by inducing protective autophagy [8,19]. This cellular recycling mechanism is believed to eliminate pathogenic protein aggregates and damaged cell components [49] and as a result may protect higher-order brain functions. In aging fruit flies, for example, the memory-promoting effects of SPD appear to be mediated via the recuperation of autophagy [11,47] and subsequent digestion of proteotoxic aggregates. More immediate effects of SPD administration might include proposed neuromodulatory actions [8]. Second, SPD mitigates age-related memory decline by inhibiting detrimental changes in presynaptic active zone dimensions and neurotransmitter release [47]. Third, disbalances in the polyamine metabolism have been described in neurological diseases [50,51], and increased dietary intake may correct these. In sum, although the exact molecular mechanisms of exogenous SPD remain partly elusive across different cell types and experimental settings [52], known molecular targets (e.g., eIF5A, histone acetyltransferases) and affected cellular processes (e.g., autophagy, mitochondrial function, translation) may converge on improved cognitive function during aging.

Additionally, our study explored the link between dietary SPM and PUT intake and dementia risk among older adults, a previously largely overlooked question. We found that higher SPM intake correlated with a reduced risk of all-cause and cause-specific dementia. Prior research has shown that long-time administration of polyamines (SPD and SPM) delays brain aging and improves cognitive function in SAMP8 mice [19]. Similar findings were observed in this study regarding the relationship between PUT intake and dementia risk. Given the scarcity of epidemiological studies exploring the effects of dietary SPM and PUT intake on dementia risk in the elderly population, further research is necessary to confirm these results.

Our prospective cohort study also examined genetic influence, including APOE genotype and polygenic risk, on the association between dietary SPD, SPM and PUT intake and dementia risk. We did not find a significant modification effect of genetic predisposition. The impact of dietary polyamines on dementia risk was independent of genetic risk. Currently, no established guidelines exist for the recommended daily intake of dietary SPD, SPM and PUT. We observed a U-shaped relationship between dietary SPD, SPM and PUT intake and dementia risk. This underscores the importance of optimizing dietary SPD (10.0–12.3 mg/day), SPM (4.4–5.7 mg/day) and PUT (13.4–17.8 mg/day) intake for dementia prevention. The associations found in our study were relatively robust in that they were significant across a multitude of subgroup analyses and sensitivity analyses so that we could propose dietary supplementation strategies for the prevention of dementia in the elderly.

Our study has several strengths. The large sample size provided enough power to examine the relationship between dietary SPD, SPM and PUT intake and various dementia outcomes, facilitating subgroup analyses. Additionally, our study defined genetic risk for dementia using both APOE genotype and a comprehensive polygenic risk score. Our findings are, furthermore, in line with previous smaller cohort studies, RCTs and preclinical data. However, several limitations of this study must be acknowledged. Firstly, although excluding participants who developed dementia within the first two or five years of follow-up yielded no evidence of reverse causality, the dementia population may have declined dietary quality earlier before their diagnoses, considering the long-time course of the preclinical phase [53,54]. Secondly, our dietary polyamine intake was calculated using Oxford WebQ, a self-reported questionnaire featuring a predetermined list of foods and beverages. However, dietary intake reported in the WebQ for the previous 24 h may not fully reflect changing habitual intake over longer periods, and there is a risk of omission for foods not included in the fixed list [55]. Thirdly, despite participants in the UK Biobank exhibiting better health and higher socioeconomic status compared to the general UK population, it is improbable that the validity of widely generalizable exposure–disease correlations would be compromised [56]. Fourth, in this study it was not possible to account for potential differences in polyamine content due to seasonal variation, preparation techniques, storage and regional variations [57]. Lastly, our study participants were limited to European ancestry, potentially constraining the generalizability of the findings to other ethnic groups.

## 5. Conclusions

This large population-based study suggests that optimal intake of dietary SPD, SPM and PUT is associated with a reduced risk of all-cause dementia, AD and VD, independent of genetic risk. Our findings highlight the importance of dietary polyamine intake in future dementia prevention strategies, regardless of genetic predisposition.

## Figures and Tables

**Table 1:** Baseline characteristics of UK Biobank participants by dietary spermidine, spermine and putrescine quintiles.

	Total (N = 77,092)	Spermidine Intake ^a^						Spermine Intake ^b^						Putrescine Intake ^c^					
		Q1 (N = 15,419)	Q2 (N = 15,418)	Q3 (N = 15,418)	Q4 (N = 15,418)	Q5 (N = 15,419)	*p* value	Q1 (N = 15,419)	Q2 (N = 15,418)	Q3 (N = 15,418)	Q4 (N = 15,418)	Q5 (N = 15,419)	*p* value	Q1 (N = 15,419)	Q2 (N = 15,418)	Q3 (N = 15,418)	Q4 (N = 15,418)	Q5 (N = 15,419)	*p* Value
Age, mean (SD), y	63.9 (2.8)	63.8 (2.8)	63.8 (2.8)	63.8 (2.8)	63.9 (2.8)	63.9 (2.8)	<0.001	63.8 (2.8)	63.8 (2.8)	63.8 (2.8)	63.9 (2.8)	63.9 (2.8)	<0.001	63.8 (2.8)	63.8 (2.8)	63.8 (2.8)	63.8 (2.8)	63.9 (2.8)	<0.001
Sex, female, n (%)	39,427 (51.1)	8835 (57.3)	8371 (54.3)	7935 (51.5)	7465 (48.4)	6821 (44.2)	<0.001	8835 (57.3)	8371 (54.3)	7935 (51.5)	7465 (48.4)	6821 (44.2)	<0.001	8388 (54.4)	8009 (51.9)	7829 (50.8)	7846 (50.9)	7355 (47.7)	<0.001
Socioeconomic status, n (%)							<0.001						<0.001						<0.001
1st quintile (least deprived)	15,420 (20.0)	2795 (18.1)	3100 (20.1)	3152 (20.4)	3181 (20.6)	3192 (20.7)		2795 (18.1)	3100 (20.1)	3152 (20.4)	3181 (20.6)	3192 (20.7)		2856 (18.5)	3156 (20.5)	3248 (21.1)	3170 (20.6)	2990 (19.4)	
2nd–4th quintiles (medium)	46,255 (60.0)	9061 (58.8)	9276 (60.2)	9307 (60.4)	9310 (60.4)	9301 (60.3)		9061 (58.8)	9276 (60.2)	9307 (60.4)	9310 (60.4)	9301 (60.3)		9110 (59.1)	9166 (59.5)	9327 (60.5)	9336 (60.6)	9316 (60.4)	
5th quintile (most deprived)	15,417 (20.0)	3563 (23.1)	3042 (19.7)	2959 (19.2)	2927 (19.0)	2926 (19.0)		3563 (23.1)	3042 (19.7)	2959 (19.2)	2927 (19.0)	2926 (19.0)		3453 (22.4)	3096 (20.1)	2843 (18.4)	2912 (18.9)	3113 (20.2)	
Education, n (%)							<0.001						<0.001						<0.001
Higher	27,977 (36.3)	5312 (34.5)	5783 (37.5)	5751 (37.3)	5615 (36.4)	5516 (35.8)		5312 (34.5)	5783 (37.5)	5751 (37.3)	5615 (36.4)	5516 (35.8)		4728 (30.7)	5516 (35.8)	5743 (37.2)	6012 (39.0)	5978 (38.8)	
Others ^d^	48,678 (63.1)	9975 (64.7)	9556 (62.0)	9598 (62.3)	9728 (63.1)	9821 (63.7)		9975 (64.7)	9556 (62.0)	9598 (62.3)	9728 (63.1)	9821 (63.7)		10,563 (68.5)	9821 (63.7)	9604 (62.3)	9333 (60.5)	9357 (60.7)	
Unknown	437 (0.6)	132 (0.9)	79 (0.5)	69 (0.4)	75 (0.5)	82 (0.5)		132 (0.9)	79 (0.5)	69 (0.4)	75 (0.5)	82 (0.5)		128 (0.8)	81 (0.5)	71 (0.5)	73 (0.5)	84 (0.5)	
Smoking status, n (%)							<0.001						<0.001						<0.001
Never	39,285 (51.0)	7817 (50.7)	8013 (52.0)	7921 (51.4)	7824 (50.7)	7710 (50.0)		7817 (50.7)	8013 (52.0)	7921 (51.4)	7824 (50.7)	7710 (50.0)		7607 (49.3)	7839 (50.8)	7897 (51.2)	7982 (51.8)	7960 (51.6)	
Previous	33,087 (42.9)	6496 (42.1)	6420 (41.6)	6621 (42.9)	6717 (43.6)	6833 (44.3)		6496 (42.1)	6420 (41.6)	6621 (42.9)	6717 (43.6)	6833 (44.3)		6499 (42.1)	6586 (42.7)	6662 (43.2)	6692 (43.4)	6648 (43.1)	
Current	4504 (5.8)	1050 (6.8)	948 (6.1)	837 (5.4)	839 (5.4)	830 (5.4)		1050 (6.8)	948 (6.1)	837 (5.4)	839 (5.4)	830 (5.4)		1256 (8.1)	963 (6.2)	816 (5.3)	700 (4.5)	769 (5.0)	
Unknown	216 (0.3)	56 (0.4)	37 (0.2)	39 (0.3)	38 (0.2)	46 (0.3)		56 (0.4)	37 (0.2)	39 (0.3)	38 (0.2)	46 (0.3)		57 (0.4)	30 (0.2)	43 (0.3)	44 (0.3)	42 (0.3)	
Alcohol intake, time(s)/week, n (%)							<0.001						<0.001						<0.001
<1	19,308 (25.0)	4350 (28.2)	3874 (25.1)	3805 (24.7)	3675 (23.8)	3604 (23.4)		4350 (28.2)	3874 (25.1)	3805 (24.7)	3675 (23.8)	3604 (23.4)		4614 (29.9)	3769 (24.4)	3549 (23.0)	3536 (22.9)	3840 (24.9)	
1–2	17,269 (22.4)	3516 (22.8)	3515 (22.8)	3360 (21.8)	3444 (22.3)	3434 (22.3)		3516 (22.8)	3515 (22.8)	3360 (21.8)	3444 (22.3)	3434 (22.3)		3583 (23.2)	3405 (22.1)	3402 (22.1)	3340 (21.7)	3539 (23.0)	
3–4	18,926 (24.5)	3624 (23.5)	3791 (24.6)	3813 (24.7)	3885 (25.2)	3813 (24.7)		3624 (23.5)	3791 (24.6)	3813 (24.7)	3885 (25.2)	3813 (24.7)		3499 (22.7)	3732 (24.2)	3877 (25.1)	3973 (25.8)	3845 (24.9)	
Daily or almost daily	21,566 (28.0)	3920 (25.4)	4232 (27.4)	4438 (28.8)	4411 (28.6)	4565 (29.6)		3920 (25.4)	4232 (27.4)	4438 (28.8)	4411 (28.6)	4565 (29.6)		3716 (24.1)	4504 (29.2)	4588 (29.8)	4564 (29.6)	4194 (27.2)	
Unknown	23 (0.0)	9 (0.1)	6 (0.0)	2 (0.0)	3 (0.0)	3 (0.0)		9 (0.1)	6 (0.0)	2 (0.0)	3 (0.0)	3 (0.0)		7 (0.0)	8 (0.1)	2 (0.0)	5 (0.0)	1 (0.0)	
Energy, mean (SD), KJ	8640 (2180)	7420 (1980)	8240 (1890)	8710 (1930)	9150 (2030)	9660 (2310)	<0.001	7420 (1980)	8240 (1890)	8710 (1930)	9150 (2030)	9660 (2310)	<0.001	7570 (2020)	8370 (1960)	8790 (2020)	9070 (2110)	9380 (2290)	<0.001
Physical activity levels ^e^, n (%)							<0.001						0.021						<0.001
Low (least active)	66,754 (86.6)	13,348 (86.6)	13,387 (86.8)	13,450 (87.2)	13,302 (86.3)	13,267 (86.0)		13,348 (86.6)	13,387 (86.8)	13,450 (87.2)	13,302 (86.3)	13,267 (86.0)		13,510 (87.6)	13,432 (87.1)	13,432 (87.1)	13,322 (86.4)	13,058 (84.7)	
High (most active)	10,338 (13.4)	2071 (13.4)	2031 (13.2)	1968 (12.8)	2116 (13.7)	2152 (14.0)		2071 (13.4)	2031 (13.2)	1968 (12.8)	2116 (13.7)	2152 (14.0)		1909 (12.4)	1986 (12.9)	1986 (12.9)	2096 (13.6)	2361 (15.3)	
Typical sleep duration, hours, n (%)							<0.001						<0.001						<0.001
<7	16,111 (20.9)	3423 (22.2)	3230 (20.9)	3074 (19.9)	3115 (20.2)	3269 (21.2)		3423 (22.2)	3230 (20.9)	3074 (19.9)	3115 (20.2)	3269 (21.2)		3351 (21.7)	3098 (20.1)	3205 (20.8)	3130 (20.3)	3327 (21.6)	
7–8	54,494 (70.7)	10,623 (68.9)	10,855 (70.4)	11,088 (71.9)	11,045 (71.6)	10,883 (70.6)		10,623 (68.9)	10,855 (70.4)	11,088 (71.9)	11,045 (71.6)	10,883 (70.6)		10,652 (69.1)	10,983 (71.2)	10,928 (70.9)	11,025 (71.5)	10,906 (70.7)	
>8h	6487 (8.4)	1373 (8.9)	1333 (8.6)	1256 (8.1)	1258 (8.2)	1267 (8.2)		1373 (8.9)	1333 (8.6)	1256 (8.1)	1258 (8.2)	1267 (8.2)		1416 (9.2)	1337 (8.7)	1285 (8.3)	1263 (8.2)	1186 (7.7)	
Polygenic risk category ^f^, n (%)							0.046						0.436						0.017
Low	15,419 (20.0)	3030 (19.7)	3148 (20.4)	3038 (19.7)	3145 (20.4)	3058 (19.8)		3030 (19.7)	3148 (20.4)	3038 (19.7)	3145 (20.4)	3058 (19.8)		3133 (20.3)	3175 (20.6)	3024 (19.6)	3104 (20.1)	2983 (19.3)	
Middle	46,254 (60.0)	9232 (59.9)	9200 (59.7)	9289 (60.2)	9218 (59.8)	9315 (60.4)		9232 (59.9)	9200 (59.7)	9289 (60.2)	9218 (59.8)	9315 (60.4)		9171 (59.5)	9172 (59.5)	9266 (60.1)	9359 (60.7)	9286 (60.2)	
High	15,419 (20.0)	3157 (20.5)	3070 (19.9)	3091 (20.0)	3055 (19.8)	3046 (19.8)		3157 (20.5)	3070 (19.9)	3091 (20.0)	3055 (19.8)	3046 (19.8)		3115 (20.2)	3071 (19.9)	3128 (20.3)	2955 (19.2)	3150 (20.4)	
Number of APOE *ε*4 alleles, n (%)							0.068						0.457						0.004
Zero	55,778 (72.4)	11,247 (72.9)	11,155 (72.4)	11,115 (72.1)	11,199 (72.6)	11,062 (71.7)		11,247 (72.9)	11,155 (72.4)	11,115 (72.1)	11,199 (72.6)	11,062 (71.7)		11,299 (73.3)	11,135 (72.2)	11,250 (73.0)	11,035 (71.6)	11,059 (71.7)	
One	19,668 (25.5)	3834 (24.9)	3937 (25.5)	3977 (25.8)	3895 (25.3)	4025 (26.1)		3834 (24.9)	3937 (25.5)	3977 (25.8)	3895 (25.3)	4025 (26.1)		3787 (24.6)	3928 (25.5)	3865 (25.1)	4046 (26.2)	4042 (26.2)	
Two	1646 (2.1)	338 (2.2)	326 (2.1)	326 (2.1)	324 (2.1)	332 (2.2)		338 (2.2)	326 (2.1)	326 (2.1)	324 (2.1)	332 (2.2)		333 (2.2)	355 (2.3)	303 (2.0)	337 (2.2)	318 (2.1)	
Hypertension, n (%)	25,456 (33.0)	4962 (32.2)	4966 (32.2)	5038 (32.7)	5216 (33.8)	5274 (34.2)	<0.001	4962 (32.2)	4966 (32.2)	5038 (32.7)	5216 (33.8)	5274 (34.2)	<0.001	5073 (32.9)	5030 (32.6)	5097 (33.1)	4963 (32.2)	5293 (34.3)	0.001
Hypercholesteremia, n (%)	13,590 (17.6)	2654 (17.2)	2637 (17.1)	2638 (17.1)	2754 (17.9)	2907 (18.9)	<0.001	2654 (17.2)	2637 (17.1)	2638 (17.1)	2754 (17.9)	2907 (18.9)	<0.001	2870 (18.6)	2710 (17.6)	2649 (17.2)	2617 (17.0)	2744 (17.8)	0.002
Diabetes, n (%)	4147 (5.4)	752 (4.9)	758 (4.9)	765 (5.0)	865 (5.6)	1007 (6.5)	<0.001	752 (4.9)	758 (4.9)	765 (5.0)	865 (5.6)	1007 (6.5)	<0.001	878 (5.7)	811 (5.3)	770 (5.0)	803 (5.2)	885 (5.7)	0.013
Body mass index ^g^, n (%)							<0.001						<0.001						<0.001
Normal	26,122 (33.9)	4868 (31.6)	5346 (34.7)	5443 (35.3)	5399 (35.0)	5066 (32.9)		5444 (35.3)	5429 (35.2)	5375 (34.9)	5072 (32.9)	4802 (31.1)		4969 (32.2)	5263 (34.1)	5356 (34.7)	5424 (35.2)	5110 (33.1)	
Overweight	34,729 (45.0)	7063 (45.8)	6933 (45.0)	6964 (45.2)	6960 (45.1)	6809 (44.2)		6819 (44.2)	6939 (45.0)	6867 (44.5)	7017 (45.5)	7087 (46.0)		6964 (45.2)	6971 (45.2)	6998 (45.4)	6882 (44.6)	6914 (44.8)	
Obese	16,241 (21.1)	3488 (22.6)	3139 (20.4)	3011 (19.5)	3059 (19.8)	3544 (23.0)		3156 (20.5)	3050 (19.8)	3176 (20.6)	3329 (21.6)	3530 (22.9)		3486 (22.6)	3184 (20.7)	3064 (19.9)	3112 (20.2)	3395 (22.0)	
Depression, n (%)	937 (1.2)	203 (1.3)	153 (1.0)	174 (1.1)	188 (1.2)	219 (1.4)	0.007	199 (1.3)	185 (1.2)	156 (1.0)	188 (1.2)	209 (1.4)	0.072	209 (1.4)	189 (1.2)	147 (1.0)	185 (1.2)	207 (1.3)	0.009
Stroke, n (%)	92 (0.1)	28 (0.2)	16 (0.1)	15 (0.1)	17 (0.1)	16 (0.1)	0.173	25 (0.2)	20 (0.1)	15 (0.1)	13 (0.1)	19 (0.1)	0.315	27 (0.2)	14 (0.1)	12 (0.1)	19 (0.1)	20 (0.1)	0.113

^a^ Q1: 0.088312–6.498700 mg/d, Q2: 6.498800–8.332662 mg/d, Q3: 8.332663–10.019219 mg/d, Q4: 10.019220–12.280556 mg/d, Q5: 12.280557–46.762417 mg/d. ^b^ Q1: 0.0263029–2.6796181 mg/d, Q2: 2.6796181–3.5185592 mg/d, Q3: 3.5185592–4.3835294 mg/d, Q4: 4.3835294–5.6813900 mg/d, Q5: 5.6813900–12.6166159 mg/d. ^c^ Q1: 0.058179–7.550910 mg/d, Q2: 7.550910–10.430071 mg/d, Q3: 10.430071–13.411120 mg/d, Q4: 13.411120–17.810156 mg/d, Q5: 17.810156–40.360663 mg/d. ^d^ Other qualifications indicate without a college or university level degree. ^e^ Low: <1200 (metabolic equivalent task (MET) minutes per week for all activity) and high (≥1200 MET min/week). ^f^ Polygenic risk category, low: 1st quintile, medium: 2–4, high: 5th quintile. ^g^ Body mass index, normal: BMI < 25 kg/m^2^; overweight: 25–30 kg/m^2^; obese ≥ 30 kg/m^2^.

## Data Availability

Researchers can apply to use the UK Biobank resource and access the data used. No additional data are available.

## Supplementary Materials

The following supporting information can be downloaded at: https://www.mdpi.com/article/10.3390/nu16162774/s1, Figure S1. Flow chart of the study population. Table S1. Nutrient database for polyamine intake. Table S2. Disease definitions used in the UK Biobank study. Table S3. Association of dietary polyamine with all-cause dementia, and cause-specific dementia (Alzheimer’s disease and vascular dementia) (incidence rate). Table S4. Relationship between dietary spermidine, spermine and putrescine with all-cause dementia, cause-specific dementia (Alzheimer’s disease and vascular dementia) excluding participants with less than 2 years of follow up. Table S5. Relationship between dietary spermidine, spermine and putrescine with all-cause dementia, cause-specific dementia (Alzheimer’s disease and vascular dementia) excluding participants with less than 5 years of follow up. Table S6. Relationship between dietary spermidine, spermine and putrescine with all-cause dementia, cause-specific dementia (Alzheimer’s disease and vascular dementia) excluding participants with one follow-up. Table S7. Relationship between dietary spermidine, spermine and putrescine with all cause dementia, cause-specific dementia (Alzheimer’s disease and vascular dementia) excluding participants with the top 5% and bottom 5% of dietary spermidine, spermine, putrescine. Table S8. Relationship between dietary spermidine, spermine and putrescine with all-cause dementia, cause-specific dementia (Alzheimer’s disease and vascular dementia) excluding participants with the top 10% and bottom 10% of dietary spermidine, spermine, putrescine. Table S9. Sensitivity analyses adjusting for potential effect mediators (Depression). Table S10. Sensitivity analyses adjusting for potential effect mediators (BMI). Table S11. Sensitivity analyses adjusting for potential effect mediators (Stroke).
