# Supplementary material for: Non-Linear Association of Dietary Polyamines with the Risk of Incident Dementia: Results from Population-Based Cohort of the UK Biobank
Source: Nutrients. 2024 Aug 20;16(16):2774. doi: 10.3390/nu16162774 (PMC11357304; doi:10.3390/nu16162774)
Supplement: Supplementary file 1 [file nutrients-16-02774-s001.zip › nutrients-3135580-supplementary.pdf]

Supplementary Materials
CONTENTS

Figure S1. Flow chart of the study population. 2
Table S1. Nutrient database for polyamine intake 3
Table S2. Disease definitions used in the UK Biobank study 11
Table S3. Association of dietary polyamine with all-cause dementia, and cause-specific dementia (Alzheimer’s disease and vascular dementia) (incidence rate). 14
Table S4. Relationship between dietary spermidine, spermine and putrescine with all-cause dementia, cause-specific dementia (Alzheimer’s disease and vascular dementia) excluding participants with less than 2 years of follow up. 15
Table S5. Relationship between dietary spermidine, spermine and putrescine with all-cause dementia, cause-specific dementia (Alzheimer’s disease and vascular dementia) excluding participants with less than 5 years of follow up. 16
Table S6. Relationship between dietary spermidine, spermine and putrescine with all-cause dementia, cause-specific dementia (Alzheimer’s disease and vascular dementia) excluding participants with one follow-up. 17
Table S7. Relationship between dietary spermidine, spermine and putrescine with all cause dementia, cause-specific dementia (Alzheimer’s disease and vascular dementia) excluding participants with the top 5% and bottom 5% of dietary spermidine, spermine, putrescine. 18
Table S8. Relationship between dietary spermidine, spermine and putrescine with all-cause dementia, cause-specific dementia (Alzheimer’s disease and vascular dementia) excluding participants with the top 10% and bottom 10% of dietaryspermidine, spermine, putrescine. 19
Table S9. Sensitivity analyses adjusting for potential effect mediators (Depression). 20
Table S10. Sensitivity analyses adjusting for potential effect mediators (BMI). 21
Table S11. Sensitivity analyses adjusting for potential effect mediators (Stroke). 22

Figure S1. Flow chart of the study population.

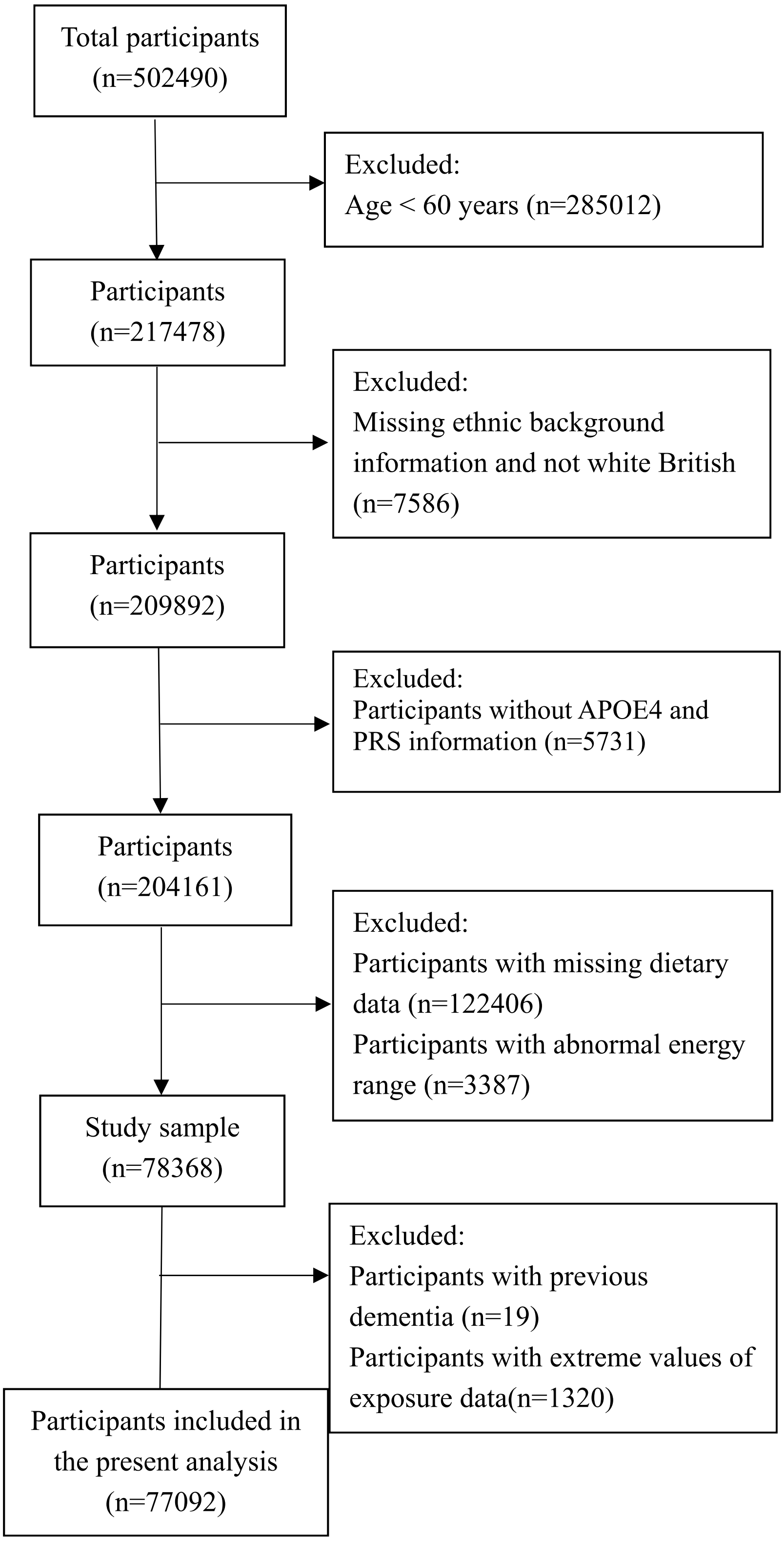

Table S1. Nutrient database for polyamine intake

Nutrient database for polyamine intake				
Food Category	Spermidine	Spermine	Putrescine	Reference
Drinks				
Water	0	0	0	
Cola	BDL	BDL	BDL	[1]
Squash	0.7	0.3	28.4	[2]
Orange soft drink	0.7	0.3	28.4	[2]
Orange juice	6	BDL	159.8	[3]
Grapefruit juice	6	BDL	99.2	[3]
Pure fruit/vegetable juice	6.8	1.0	15.9	[4]
Apple juice	2	BDL	11.1	[4]
Grape juice	5.1	BDL	9	[4]
Apricot juice	7.0	BDL	8	[4]
Tomato juice	13.2	1	35.5	[4]
Fruit smoothie	NA	NA	NA	
Dairy smoothie	NA	NA	NA	
Coffee	BDL	BDL	BDL	[3]
Tea	BDL	BDL	BDL	[3]
Other non-alcoholic drinks				
Full cream milk	2	2	1	[5]
Semi-skimmed milk	0.4	0.4	0.3	[3]
Skimmed milk	BDL	BDL	BDL	[3]
Powdered milk	7	7	1.5	[3]
Soybean milk	112	14	24	[1]
Goat/sheep milk	21.0	2.8	3.1	[2]
Ovine milk	2.1	2.5	0.5	[2]
Caprine milk	39.9	3.0	5.7	[2]
Other type of milk	3	6	0.5	[1]
Human milk	3	6	0.5	[1]
Hot chocolate	21.5	3	9	[3]
Other drink	NA			
Barley cup	NA	NA	NA	
Other mixed fruit drink	NA	NA	NA	
Alcohol				
Red wine	2	BDL	39.1	[3]
Rose wine	0.7	BDL	68.1	[6]
White wine	1	BDL	9.0	[3]
Beer/cider	BDL	BDL	21.2	[3]
Fortified wine	1	BDL	BDL	[4]
Cognac	1	BDL	BDL	[4]
Spirits	1	BDL	BDL	[4]

Whisky	1	BDL	BDL	[4]
Other alcohol	BDL	BDL	35.4	[4]
Liqueur wine	BDL	BDL	35.4	[4]
Breakfast cereal				
Breakfast cereals, mixed	166.6	31.6	113.4	[7]
Porridge	BDL	BDL	BDL	[3]
Bran cereal	351.1	438.9	553.6	[7]
Whole-wheat cereal	168	40	8	[8]
Breads	78.8	17.2	29.6	[4]
White bread	54.2	13.2	13.0	[4]
Oat bread	65.4	12.6	22.1	[4]
Rye bread	86.1	20.7	42.5	[4]
Whole bread	109.3	22.4	40.6	[4]
Oatcakes	65.4	12.6	22.1	[3]
Pastry	BDL	BDL	BDL	[9]
Crumble	NA	NA	NA	
Pizza	35.1	6.0	13	[3]
Pancake	5	1	BDL	[3]
Yorkshire pudding	NA	NA	NA	
Indian snacks	NA	NA	NA	
Croissant	NA	NA	NA	
Scone	NA	NA	NA	
Yogurt/ice-cream				
Yogurt	1	1	BDL	[3]
Ice-cream	BDL	BDL	BDL	[9]
Dessert				
Milk-based pudding	NA	NA	NA	
Other milk-based pudding	NA	NA	NA	
Soya dessert	NA	NA	NA	
Cake	6	1	3	[3]
Doughnut	NA	NA	NA	
Sponge pudding	20	2	10	[10]
Cheesecake	NA	NA	NA	
Other dessert	11.1	2	4	[3]
Strawberry pie	13.2	3	5	[3]
Lemon pie	9	1	3	[3]
Sweet snack				
Milk chocolate	6	1	3	[3]
Dark chocolate	21.5	3	9	[3]
Raisin	3	1	2	[3]
Chocolate	17	7	4	[11]
Sweets	0	0	0	[11]
Sweet biscuits	6	1	2	[3]
Cereal bar	NA	NA	NA	

Savoury snack				
Peanuts	388.7	34.6	61.4	[3]
Nuts	163.2	133.4	57.9	[1,3,12]
Almonds	207.4	54.2	35.6	[3]
Cashews	124	363	45	[1]
Pistachio	208.6	54.3	87.4	[3]
Walnuts	112.9	61.9	63.5	[12]
Seeds	225.4	64.3	57.9	[1,3]
Sesame	126	22	29	[1]
Hazelnut	144.5	32.3	47.7	[3]
Sunflower seed	383	89	34	[1]
Pumpkin seed	248	114	121	[1]
Crisp	13	245	171	[11]
Biscuits	6	1	2	[3]
Olives	NA	NA	NA	
Other savoury snack	NA	NA	NA	
Soup	95.4	22.0	31.8	[3]
Vegetable soup	35.7	5	24.5	[3]
Lentil soup	151.5	36.5	38.5	[13]
Starchy food				
Pasta	35.1	6	13	[3]
White rice	BDL	BDL	1	[3]
Brown rice	44.1	49.4	56.7	[7]
Sushi	NA	NA	NA	
Snackpot	NA	NA	NA	
Couscous	NA	NA	NA	
Other grain	42.1	29.5	34.0	[1,3]
Barley	16	20	17	[1]
Semolina	5.0	23.1	25.1	[3]
Soft wheat	105.3	45.4	60.0	[3]
Cheese				
Cheese	1.4	0.1	0.4	[3]
Hard cheese	53.7	17.3	987.0	[7]
Soft cheese	1.4	0.1	1.7	[7]
Blue cheese	111.5	7.4	475.3	[7]
Cottage cheese	NA	NA	NA	
Mozzarella	BDL	BDL	BDL	[1]
Goat's cheese	4.2	1.6	7.2	[3]
Egg				
Whole egg	BDL	BDL	20.5 ± 0.1	[3]
Scotch egg	BDL	BDL	20.5 ± 0.1	[3]
Omelette	NA	NA	NA	
Other egg	NA	NA	NA	
Meat				

Sausage	20.0	46.8	211.0	[3]
Toulouse sausage	16.3	46.2	3.0	[3]
Spicy sausage (merguez)	12.2	48.3	8.1	[3]
Frankfurter sausage	27.0	31.1	11.1	[3]
Garlic sausage	31.5	45.5	7	[3]
Rosette (gamy pork sausage)	13.0	62.9	1025.6	[3]
Beef	17.5	140.8	30.5	[3]
Pork	9.3	72.3	4	[3]
Lamb	39.7	131.3	8.2	[3]
Poultry	91.1	65.6	168.0	[2,3]
Chicken	78.2	61.4	10.1	[3]
Turkey	103.9	69.8	325.8	[2,3]
Chicken skin	78.5	120.1	0.0	[2]
Bacon	6	26.2	2	[3]
Ham	8.1	63.4	5	[3]
Liver	199.7	509.0	26.1	[6]
Other meat	82	305	135.5	[1,14]
Duck	58	323	20	[1]
Game	106	287	251	[14]
Fish				
Oily fish	45.1	47.6	35.7	[1-3]
Salmon	60.0	45.0	44.0	[3]
Mackerel	49	92	20.0	[1]
Herring	26.2	5.9	43.1	[2]
Shrimp	2.4	BDL	2.7	[3]
Lobster/crab	4.0	BDL	1	[3]
Shellfish	93.6	64.6	261.6	[3]
Scallops (coral)	14.5	49.9	487.3	[3]
Scallops (white)	6.8	14.0	285.7	[3]
Muscles	259.5	129.8	11.7	[3]
Other fish	19.8	51.0	10.2	[1,7]
Trout	27.5	44.0	20.4	[7]
Squid	12	58	BDL	[1]
Tinned tuna	6	10	2	[3]
White fish	160	75	119	[1]
Vegetarian alternatives				
Vegetarian sausages/burgers	NA	NA	NA	
Tofu	107	32	20	[1]
Quorn	NA	NA	NA	
Other vegetarian alternative	134.1	23.9	30.8	[3]
Nut roast	268.2	47.7	61.5	[3]
Almond (grilled nut)	207.4	54.2	35.6	[3]
Pistachio (grilled nut)	208.6	54.3	87.4	[3]
Peanut (grilled nut)	388.7	34.6	61.4	[3]

Falafel	NA	NA	NA	
	Vegetable			
Cooked bean	57.1	24.7	55.6	[7]
Pulses	165.4	45.7	30.7	[3,14]
Kidney beans	134	120	4	[14]
Chick peas	198.3	6	29.4	[3]
Lentils	163.9	11.1	58.7	[3]
Cooked potatoes	105.0	26.0	245.0	[5]
Butter	BDL	BDL	BDL	[3]
Potato	77.0	15.0	110.0	[5]
Mashed potato	120.7	2	87.4	[3]
Mixed vegetable	106.3	14.0	39.1	[3]
Cabbage	195.3	18.1	74.9	[3]
Avocado	12.7	6.6	3.1	[3]
Broad bean	495.7	118.6	203.1	[12]
Green bean	74.4	13.3	116.8	[12]
Beetroot	29.1	BDL	51.7	[3]
Broccoli	214.0	22.8	64.2	[3]
Cabbage/kale	195.3	8.1	74.9	[3]
Carrot	55.0	12.0	17.0	[5]
Cauliflower	56.0	43.0	171.0	[5]
Celery	22.0	0.7	25.0	[3]
Courgette	107.0	7.5	393.5	[3]
Cucumber	65.0	1.3	98.9	[3]
Garlic	155.3	33.8	13.1	[3]
Leek	104.7	10.7	27.4	[3]
Leek (green)	127.2	12.3	30.2	[3]
Leek (white)	82.2	9.1	24.6	[3]
Lettuce	95.6	5.9	89.4	[3]
Mushroom	450.0	3.0	4.0	[3]
Onion	35.45	BDL	5.7	[3]
Radish	87.6	1.7	10.5	[3]
Pea	348.4	19.1	196.5	[3]
Sweet pepper	32.4	8.4	26.9	[3]
Spinach	185	18	50	[1]
Sprouts	127.6	9.1	59.1	[3]
Corn	298	6	520	[1]
Sweet potato	20	29	15	[1]
Fresh tomato	19.4	BDL	381.1	[3]
Tinned tomato	34.2	1	163.1	[3]
Turnip	405	19	219	[1]
Watercress	201	7	20	[1]
Other vegetables	82.7	48.7	116.7	[1,11]
Aubergine	86	27	214	[1]

Asparagus	120	28	61	[11]
Pumpkin	42	91	75	[11]
Fruit				
Stewed fruit				
Apple stew	7	BDL	5	[3]
Prune	10.1	2	6.1	[3]
Dried fruit	8.0	0.7	15.4	[1,3]
Raisins	3	1	2	[3]
Dates	10.1	1	32.2	[3]
Prune (dried)	11	0	12	[1]
Mixed fruit	15.3	1	27.6	[3]
Apple	5-17	0	12	[5]
Banana	44.9	1	317.3	[3]
Berry	37.7	6.9	14.8	[1,3]
Strawberry	40.3	13.7	18.5	[3]
Blueberry	35	BDL	11	[1]
Cherry	19	BDL	53	[1]
Grapefruit	19.5	1.5	436	[1]
White grapefruit	19	3	292	[1]
Ruby grapefruit	20	BDL	580	[1]
Grape	22.5	1.6	26.3	[3]
Grape(red)	15.2	0.1	34.2	[3]
Grape(green)	29.8	3.1	18.3	[3]
Mango	206.5	15.8	907.5	[7]
Melon	77.7	4.9	22.7	[3]
Orange	17.8	BDL	1047.7	[3]
Satsuma	80	BDL	860	[1]
Peach	18.4	BDL	7.4	[3]
Pear	18.4	0.2	0.4	[3]
Pineapple	27.0	10.9	7.6	[3]
Plum	11.7	0.5	30.6	[12]
Other fruit	31.2	1.7	20.0	[1,3,12]
Kiwi	37.3	7.4	13.3	[3]
Papaya	37	BDL	53	[1]
Pomegranate	5	BDL	2	[1]
Fig	36	BDL	25	[1]
Apricot	40.6	1.0	6.8	[12]
Seasonings				
Salt	BDL	BDL	BDL	[3]
Sugar	0	0	0	[11]
Artificial sweetener	NA	NA	NA	NA

Values expressed in nmol/g ± SD or ml ± SD or mean (range); BDL, below detection limits; NA, not available;

References:

1 K. Nishimura, R. Shiina, K. Kashiwagi, K. Igarashi. Decrease in polyamines with aging and their ingestion from food and drink. J Biochem, 2006, **139**(1)**:** 81-90.

2 P. Kalac. Health effects and occurrence of dietary polyamines: a review for the period 2005-mid 2013. Food Chem, 2014, **161:** 27-39.

3 B. G. Cipolla, R. Havouis, J. P. Moulinoux. Polyamine contents in current foods: a basis for polyamine reduced diet and a study of its long term observance and tolerance in prostate carcinoma patients. Amino Acids, 2007, **33**(2)**:** 203-212.

4 P. Kalac, Martin Krizek, Tamara Pelikanova, Marketa Langova, O. %J Food Chemistry Veskrna. Contents of polyamines in selected foods. 2005, **90**(4)**:** 561-564.

5 S Bardócz, G. Grant, D. S. Brown, A. Ralph, A. %J J.nutr.biochem Pusztai. Polyamines in food—implications for growth and health. 1993, **4**(2)**:** 66-71.

6 R. Romero, M. Sánchez-Viñas, D. Gázquez, M. G. Bagur. Characterization of selected Spanish table wine samples according to their biogenic amine content from liquid chromatographic determination. J Agric Food Chem, 2002, **50**(16)**:** 4713-4717.

7 M. Atiya Ali, E. Poortvliet, R. Stromberg, A. Yngve. Polyamines in foods: development of a food database. Food Nutr Res, 2011, **55**.

8 Knut A Eliassen, Ragnhild Reistad, Unni Risøen, Helle F Rønning. Dietary polyamines. Food Chemistry, 2002, **78**(78)**:** 273-280.

9 S. Kiechl, R. Pechlaner, P. Willeit, M. Notdurfter, B. Paulweber, K. Willeit, P. Werner, C. Ruckenstuhl, B. Iglseder, S. Weger, B. Mairhofer, M. Gartner, L. Kedenko, M. Chmelikova, S. Stekovic, H. Stuppner, F. Oberhollenzer, G. Kroemer, M. Mayr, T. Eisenberg, H. Tilg, F. Madeo, J. Willeit. Higher spermidine intake is linked to lower mortality: a prospective population-based study. Am J Clin Nutr, 2018, **108**(2)**:** 371-380.

10 P. Krausová, P. Kalač, M. Křížek, T. Pelikánová. Content of biologically active polyamines in livers of cattle, pigs and chickens after animal slaughter. Meat Sci, 2006, **73**(4)**:** 640-644.

11 Naoyoshi Nishibori, Shinsuke Fujihara, Toshiko Akatuki. Amounts of polyamines in foods in Japan and intake by Japanese. Food Chemistry, 2007, **100**(2)**:** 491-497.

12 Nelly C. Muñoz-Esparza, Judit Costa-Catala, Oriol Comas-Basté, Natalia Toro-Funes, M. Luz Latorre-Moratalla, M. Teresa Veciana-Nogués, M. Carmen Vidal-Carou. Occurrence of Polyamines in Foods and the Influence of Cooking Processes. 2021, **10**(8)**:** 1752.

13 S. Bardocz, T. J. Duguid, D. S. Brown, G. Grant, A. Pusztai, A. White, A. Ralph. The importance of dietary polyamines in cell regeneration and growth. Br J Nutr, 1995, **73**(6)**:** 819-828.

14 Pavel Kalač, Petra Krausová. A review of dietary polyamines: Formation,

implications for growth and health and occurrence in foods. Food Chemistry, 2005, **90**(1–2)**:** 219-230.

Table S2. Disease definitions used in the UK Biobank study

	ICD-9	ICD-10	Self-reported in UK Biobank (field ID)	Reference
All-cause dementia	2902, 2903, 2904, 2912, 2941, 3310, 3311, 3312, 3315	A810, F00, F000, F001, F002, F009, F01, F010, F011, F012, F013, F018, F019, F02, F020, F021, F022, F023, F024, F028, F03, F051, F106, G30, G300, G301, G308, G309, G310, G311, G318, I673	20002(1263)	[1] [2] [3] [4]
Alzheimer's disease	3310	F00, F000, F001, F002, F009, G30, G300, G301, G308, G309,		[2] [3] [4]
Vascular dementia	2904	F01, F010, F011, F012, F013, F018, F019, I673		[2] [3] [4]
Coronary Heart Disease (CHD)	410, 411, 412, 413, 414	I20, I21, I22, I23, I24, I25	6150(1), 20002(1075, 1074), 20004(1070, 1095, 1523)	[5] [6] [7] [8]
Diabetes	25000, 25001, 25009, 25010, 25011, 25019, 25029, 2503, 2504, 2505, 25099	E10, E100, E101, E102, E103, E104, E105, E106, E107, E108, E109, E11, E110, E111, E112, E113, E114, E115, E116, E117,E118, E119, E12, E120, E121, E122, E123, E124, E125, E126, E127, E128, E129, E13, E130, E131, E132, E133, E134, E135, E136, E137, E138, E139, E14, E140, E141, E142, E143, E144, E145, E146, E147, E148, E149, E15, E150, E151, E152, E153, E154, E155, E156, E157, E158, E159	20002(1220, 1222, 1223), 2443(1), 6153(3), 6177(3)	[9] [10] [11] [4]
Depression	2962, 2963, 3004, 311	F32, F33, F341, F381, F39, F204	20002(1286, 1531)	[12] [13]
Hypertension	401, 402, 403, 404, 405	I10, I11, I110, I119, I12, I120, I129, I13, I130, I131, I132, I139, I15, I150, I151, I152, I158, I159	20002(1065, 1072), 6153(2), 6177(2), 6150(4), 2966	[14] [12]
Hypercholesteremia	2720	E78	20002(1473)	[12, 15]
Stroke	3361, 36231, 36232, 430, 431, 4329, 43301, 43311, 43321, 43331, 43381, 43391, 434, 436	I60, I600, I601, I602, I603, I604, I605, I606, I607, I608, I609, I61, I610, I611, I612, I613, I614, I615, I616, I618, I619, I63, I630, I631, I632, I633, I634, I635, I636, I638, I639, I64, I678, I690, I693, G951, H341, H342, S066	6150(3), 4056, 20002(1081, 1491, 1583, 1086)	[14, 16]

References:

[1] WILKINSON T, SCHNIER C, BUSH K, et al Identifying dementia outcomes in UK Biobank: a validation study of primary care, hospital admissions and mortality data [J] European journal of epidemiology, 2019, 34(6): 557-65

[2] ZHANG H, GREENWOOD D C, RISCH H A, et al Meat consumption and risk of incident dementia: cohort study of 493,888 UK Biobank participants [J] The American journal of clinical nutrition, 2021, 114(1): 175-84

[3] HU H Y, WU B S, OU Y N, et al Tea consumption and risk of incident dementia: A prospective cohort study of 377 592 UK Biobank participants [J] Translational psychiatry, 2022, 12(1): 171

[4] TAI X Y, VELDSMAN M, LYALL D M, et al Cardiometabolic multimorbidity, genetic risk, and dementia: a prospective cohort study [J] The lancet Healthy longevity, 2022, 3(6): e428-e36

[5] GREGSON J, KAPTOGE S, BOLTON T, et al Cardiovascular Risk Factors Associated With Venous Thromboembolism [J] JAMA cardiology, 2019, 4(2): 163-73

[6] WANG M, BRAGE S, SHARP S J, et al Associations of genetic susceptibility and healthy lifestyle with incidence of coronary heart disease and stroke in individuals with hypertension [J] European journal of preventive cardiology, 2022, 29(16): 2101-10

[7] EMDIN C A, KHERA A V, NATARAJAN P, et al Genetic Association of Waist-to-Hip Ratio With Cardiometabolic Traits, Type 2 Diabetes, and Coronary Heart Disease [J] Jama, 2017, 317(6): 626-34

[8] KIM Y, YEUNG S L A, SHARP S J, et al Genetic susceptibility, screen-based sedentary activities and incidence of coronary heart disease [J] BMC medicine, 2022, 20(1): 188

[9] WANG M, ZHOU T, LI X, et al Baseline Vitamin D Status, Sleep Patterns, and the Risk of Incident Type 2 Diabetes in Data From the UK Biobank Study [J] Diabetes care, 2020, 43(11): 2776-84

[10] SONG Z, YANG R, WANG W, et al Association of healthy lifestyle including a healthy sleep pattern with incident type 2 diabetes mellitus among individuals with hypertension [J] Cardiovascular diabetology, 2021, 20(1): 239

[11] ZHOU Z, MACPHERSON J, GRAY S R, et al Are people with metabolically healthy obesity really healthy? A prospective cohort study of 381,363 UK Biobank participants [J] Diabetologia, 2021, 64(9): 1963-72

[12] SHANG X, ZHANG X, HUANG Y, et al Temporal trajectories of important diseases in the life course and premature mortality in the UK Biobank [J] BMC medicine, 2022, 20(1): 185

[13] DREGAN A, RAYNER L, DAVIS K A S, et al Associations Between Depression, Arterial Stiffness, and Metabolic Syndrome Among Adults in the UK Biobank Population Study: A Mediation Analysis [J] JAMA psychiatry, 2020, 77(6): 598-606

[14] SAID M A, VERWEIJ N, VAN DER HARST P Associations of Combined Genetic and Lifestyle Risks With Incident Cardiovascular Disease and

Diabetes in the UK Biobank Study [J] JAMA cardiology, 2018, 3(8): 693-702
[15] SHANG X, ZHU Z, ZHANG X, et al Association of a wide range of chronic diseases and apolipoprotein E4 genotype with subsequent risk of dementia in community-dwelling adults: A retrospective cohort study [J] EClinicalMedicine, 2022, 45: 101335
[16] CHO S M J, KOYAMA S, RUAN Y, et al Measured Blood Pressure, Genetically Predicted Blood Pressure, and Cardiovascular Disease Risk in the UK Biobank [J] JAMA cardiology, 2022, 7(11): 1129-37

Table S3. Association of dietary polyamine with all-cause dementia, and cause-specific dementia (Alzheimer's disease and vascular dementia) (incidence rate).

No. of cases/1,000 person-years(IR)	Dietary Spermidine					Dietary Spermine					Dietary Putrescine				
	Q1	Q2	Q3	Q4	Q5	Q1	Q2	Q3	Q4	Q5	Q1	Q2	Q3	Q4	Q5
**All-cause dementia**	257/185.4932(1.39)	198/188.581(1.05)	203/188.552(1.08)	179/188.7264(0.95)	250/187.8506(1.33)	254/186.5994(1.36)	193/188.3779(1.02)	192/188.3918(1.02)	217/188.4626(1.15)	231/187.6717(1.23)	250/186.2428(1.34)	193/188.4653(1.02)	218/188.3896(1.16)	205/188.309(1.09)	221/188.0966(1.17)
**AD**	105/184.216(0.57)	91/187.7137(0.48)	82/187.7763(0.44)	69/187.7855(0.37)	103/186.5835(0.55)	120/1854777(0.64)	73/1873874(0.39)	67/1873247(0.36)	101/187.4293(0.54)	89/186.456(0.48)	112/185.0982(0.61)	78/187.4492(0.42)	71/187.1535(0.39)	104/187.4447(0.55)	85/186.9294(0.45)
**VD**	59/183.847(0.32)	39/187.2419(0.21)	34/187.3622(0.18)	33/187.4607(0.18)	41/186.0175(0.22)	50/184.8956(0.27)	42/187.1067(0.22)	42/187.1103(0.22)	35/186.8639(0.19)	37/185.9527(0.20)	58/184.6891(0.31)	36/187.0517(0.19)	42/186.8861(0.22)	26/186.7347(0.14)	44/186.5675(0.24)

Abbreviations: AD, Alzheimer's disease; VD, vascular dementia; IR, incidence rate.

Table S4. Relationship between dietary spermidine, spermine and putrescine with all-cause dementia, cause-specific dementia (Alzheimer's disease and vascular dementia) excluding participants with less than 2 years of follow up.

Polyamines		All-cause dementia		AD		VD	
		**HR(95% CI)**	***P value***	**HR(95% CI)**	***P value***	**HR(95% CI)**	***P value***
**Dietary Spermidine**	Q1	1.00 (ref.)	-	1.00 (ref.)	-	1.00 (ref.)	-
	Q2	0.76(0.63, 0.91)	0.004	0.84(0.63, 1.12)	0.240	0.68(0.45, 1.02)	0.062
	Q3	0.78(0.64, 0.94)	0.009	0.76(0.56, 1.02)	0.065	0.56(0.36, 0.88)	0.011
	Q4	0.67(0.55, 0.82)	<0.001	0.61(0.44, 0.83)	0.002	0.57(0.36, 0.88)	0.011
	Q5	0.86(0.71, 1.04)	0.129	0.82(0.61, 1.1)	0.191	0.63(0.41, 0.97)	0.035
**Dietary Spermine**	Q1	1.00 (ref.)	-	1.00 (ref.)	-	1.00 (ref.)	-
	Q2	0.76(0.62, 0.91)	0.004	0.59(0.44, 0.8)	0.001	0.84(0.55, 1.28)	0.418
	Q3	0.73(0.60, 0.89)	0.001	0.52(0.39, 0.71)	<0.001	0.83(0.55, 1.27)	0.398
	Q4	0.83(0.68, 1.00)	0.049	0.79(0.6, 1.04)	0.095	0.67(0.43, 1.06)	0.087
	Q5	0.82(0.68, 1.00)	0.048	0.64(0.48, 0.86)	0.003	0.64(0.41, 1.02)	0.059
**Dietary Putrescine**	Q1	1.00 (ref.)	-	1.00 (ref.)	-	1.00 (ref.)	-
	Q2	0.78(0.65, 0.95)	0.012	0.69(0.51, 0.93)	0.014	0.66(0.43, 1)	0.052
	Q3	0.9(0.75, 1.09)	0.290	0.65(0.48, 0.88)	0.005	0.79(0.52, 1.19)	0.261
	Q4	0.82(0.68, 1.00)	0.048	0.9(0.68, 1.19)	0.467	0.49(0.3, 0.78)	0.003
	Q5	0.85(0.7, 1.03)	0.096	0.72(0.53, 0.97)	0.028	0.75(0.49, 1.14)	0.184

Abbreviations: HR, hazard ratio; CI, confidence interval; -: Not available; AD, Alzheimer's disease; VD, vascular dementia; Ref , reference.

Analyses were adjusted age, sex, socioeconomic status (least deprived/medium/most deprived), education (high/other), smoke status (never/previous/current), alcohol intake (<1 time/week, 1–2 times/week, 3–4times/week, daily or almost daily), energy, sleep duration (<7 hours/7–8 hours/>8 hours), physical activity levels (moderate/low/high), polygenic risk score, APOE ε4, hypertension (no/yes), hypercholesteremia (no/yes), diabetes (no/yes) and first 10 principal components of ancestry.

Table S5. Relationship between dietary spermidine, spermine and putrescine with all-cause dementia, cause-specific dementia (Alzheimer's disease and vascular dementia) excluding participants with less than 5 years of follow up.

Polyamines		All-cause dementia		AD		VD	
		HR(95% CI)	*P value*	HR(95% CI)	*P value*	HR(95% CI)	*P value*
**Dietary Spermidine**	Q1	1.00 (ref.)	-	1.00 (ref.)	-	1.00 (ref.)	-
	Q2	0.76(0.62, 0.93)	0.007	0.85(0.63, 1.15)	0.301	0.63(0.41, 0.97)	0.035
	Q3	0.81(0.67, 0.99)	0.037	0.76(0.55, 1.03)	0.076	0.55(0.35, 0.87)	0.010
	Q4	0.68(0.55, 0.84)	<0.001	0.66(0.47, 0.91)	0.011	0.55(0.35, 0.87)	0.011
	Q5	0.90(0.74, 1.10)	0.313	0.86(0.64, 1.17)	0.348	0.62(0.40, 0.97)	0.037
**Dietary Spermine**	Q1	1.00 (ref.)	-	1.00 (ref.)	-	1.00 (ref.)	-
	Q2	0.74(0.61, 0.91)	0.004	0.60(0.44, 0.81)	0.001	0.80(0.52, 1.25)	0.332
	Q3	0.74(0.60, 0.90)	0.003	0.54(0.39, 0.74)	<0.001	0.82(0.53, 1.28)	0.381
	Q4	0.84(0.69, 1.03)	0.091	0.79(0.59, 1.06)	0.110	0.71(0.45, 1.13)	0.150
	Q5	0.85(0.70, 1.04)	0.115	0.69(0.51, 0.93)	0.015	0.62(0.38, 1.00)	0.050
**Dietary Putrescine**	Q1	1.00 (ref.)	-	1.00 (ref.)	-	1.00 (ref.)	-
	Q2	0.83(0.68, 1.02)	0.072	0.76(0.56, 1.03)	0.076	0.62(0.40, 0.97)	0.038
	Q3	0.89(0.73, 1.08)	0.231	0.69(0.50, 0.95)	0.023	0.74(0.48, 1.15)	0.181
	Q4	0.84(0.69, 1.03)	0.100	0.97(0.72, 1.30)	0.823	0.50(0.31, 0.81)	0.005
	Q5	0.91(0.74, 1.10)	0.325	0.79(0.58, 1.08)	0.134	0.73(0.47, 1.13)	0.158

Abbreviations: HR, hazard ratio; CI, confidence interval; -: Not available; AD, Alzheimer's disease; VD, vascular dementia; Ref , reference.

Analyses were adjusted for age, sex, socioeconomic status (least deprived/medium/most deprived), education (high/other), smoke status (never/previous/current), alcohol intake (<1 time/week, 1–2 times/week, 3–4times/week, daily or almost daily), energy, sleep duration (<7 hours/7–8 hours/>8 hours), physical activity levels (moderate/low/high), polygenic risk score, APOE ε4, hypertension (no/yes), hypercholesteremia (no/yes), diabetes (no/yes) and first 10 principal components of ancestry.

Table S6. Relationship between dietary spermidine, spermine and putrescine with all-cause dementia, cause-specific dementia (Alzheimer's disease and vascular dementia) excluding participants with one follow-up.

Polyamines		All-cause dementia		AD		VD	
		HR(95% CI)	*P* value	HR(95% CI)	*P* value	HR(95% CI)	*P* value
**Dietary Spermidine**	Q1	1.00 (ref.)	-	1.00 (ref.)	-	1.00 (ref.)	-
	Q2	0.70(0.53, 0.91)	0.009	0.92(0.61, 1.39)	0.684	0.70(0.38, 1.27)	0.238
	Q3	0.76(0.58, 0.99)	0.044	0.80(0.52, 1.23)	0.315	0.63(0.34, 1.18)	0.151
	Q4	0.58(0.43, 0.77)	<0.001	0.61(0.38, 0.96)	0.034	0.53(0.27, 1.03)	0.060
	Q5	0.69(0.51, 0.92)	0.013	0.73(0.45, 1.16)	0.184	0.58(0.29, 1.18)	0.134
**Dietary Spermine**	Q1	1.00 (ref.)	-	1.00 (ref.)	-	1.00 (ref.)	-
	Q2	0.75(0.57, 0.98)	0.037	0.68(0.45, 1.03)	0.069	0.96(0.52, 1.77)	0.887
	Q3	0.62(0.47, 0.82)	0.001	0.48(0.31, 0.76)	0.002	0.57(0.29, 1.15)	0.115
	Q4	0.78(0.59, 1.03)	0.082	0.88(0.59, 1.33)	0.547	0.79(0.41, 1.53)	0.484
	Q5	0.72(0.54, 0.96)	0.027	0.59(0.37, 0.94)	0.027	0.60(0.28, 1.26)	0.174
**Dietary Putrescine**	Q1	1.00 (ref.)	-	1.00 (ref.)	-	1.00 (ref.)	-
	Q2	0.72(0.54, 0.95)	0.021	0.77(0.5, 1.18)	0.222	0.4(0.21, 0.75)	0.005
	Q3	0.90(0.69, 1.18)	0.461	0.69(0.44, 1.08)	0.107	0.53(0.29, 0.96)	0.036
	Q4	0.82(0.62, 1.09)	0.168	0.98(0.65, 1.48)	0.926	0.42(0.22, 0.80)	0.008
	Q5	0.81(0.61, 1.09)	0.163	0.80(0.51, 1.26)	0.342	0.49(0.26, 0.94)	0.031

Abbreviations: HR, hazard ratio; CI, confidence interval; -: Not available; AD, Alzheimer's disease; VD, vascular dementia; Ref, reference.

Analyses were adjusted for age, sex, socioeconomic status (least deprived/medium/most deprived), education (high/other), smoke status (never/previous/current), alcohol intake (<1 time/week, 1–2 times/week, 3–4times/week, daily or almost daily), energy, sleep duration (<7 hours/7–8 hours/>8 hours), physical activity levels (moderate/low/high), polygenic risk score, APOE ε4, hypertension (no/yes), hypercholesteremia (no/yes), diabetes (no/yes) and first 10 principal components of ancestry.

Table S7. Relationship between dietary spermidine, spermine and putrescine with all cause dementia, cause-specific dementia (Alzheimer's disease and vascular dementia) excluding participants with the top 5% and bottom 5% of dietary spermidine, spermine, putrescine.

**Polyamines**		**All-cause dementia**		**AD**		**VD**	
		**HR(95% CI)**	***P value***	**HR(95% CI)**	***P value***	**HR(95% CI)**	***P value***
**Dietary Spermidine**	Q1	1.00 (ref.)	-	1.00 (ref.)	-	1.00 (ref.)	-
	Q2	0.79(0.64, 0.96)	0.020	0.90(0.66, 1.22)	0.502	0.68(0.44, 1.06)	0.087
	Q3	0.80(0.65, 0.98)	0.031	0.80(0.58, 1.10)	0.165	0.59(0.38, 0.93)	0.024
	Q4	0.69(0.56, 0.85)	0.001	0.65(0.46, 0.91)	0.012	0.56(0.35, 0.89)	0.014
	Q5	0.88(0.71, 1.09)	0.232	0.91(0.65, 1.27)	0.574	0.56(0.34, 0.93)	0.025
**Dietary Spermine**	Q1	1.00 (ref.)	-	1.00 (ref.)	-	1.00 (ref.)	-
	Q2	0.76(0.62, 0.92)	0.006	0.56(0.41, 0.76)	<0.001	0.85(0.54, 1.32)	0.470
	Q3	0.74(0.60, 0.91)	0.004	0.50(0.36, 0.68)	<0.001	0.83(0.53, 1.30)	0.414
	Q4	0.84(0.68, 1.02)	0.081	0.75(0.56, 1.01)	0.055	0.68(0.42, 1.09)	0.109
	Q5	0.82(0.66, 1.02)	0.075	0.63(0.46, 0.87)	0.006	0.59(0.35, 1.01)	0.055
**Dietary Putrescine**	Q1	1.00 (ref.)	-	1.00 (ref.)	-	1.00 (ref.)	-
	Q2	0.84(0.68, 1.03)	0.098	0.7(0.51, 0.96)	0.028	0.74(0.47, 1.19)	0.214
	Q3	0.97(0.79, 1.19)	0.752	0.65(0.47, 0.90)	0.010	0.88(0.56, 1.39)	0.585
	Q4	0.89(0.72, 1.09)	0.270	0.92(0.68, 1.23)	0.561	0.54(0.32, 0.91)	0.020
	Q5	0.82(0.66, 1.03)	0.090	0.63(0.44, 0.90)	0.010	0.81(0.49, 1.33)	0.405

Abbreviations: HR, hazard ratio; CI, confidence interval; -: Not available; AD, Alzheimer's disease; VD, vascular dementia; Ref , reference.

Analyses were adjusted for age, sex, socioeconomic status (least deprived/medium/most deprived), education (high/other), smoke status (never/previous/current), alcohol intake (<1 time/week, 1–2 times/week, 3–4times/week, daily or almost daily), energy, sleep duration (<7 hours/7–8 hours/>8 hours), physical activity levels (moderate/low/high), polygenic risk score, APOE ε4, hypertension (no/yes), hypercholesteremia (no/yes), diabetes (no/yes) and first 10 principal components of ancestry.

Table S8. Relationship between dietary spermidine, spermine and putrescine with all-cause dementia, cause-specific dementia (Alzheimer's disease and vascular dementia) excluding participants with the top 10% and bottom 10% of dietaryspermidine, spermine, putrescine.

Polyamines		All-cause dementia		AD		VD	
		HR(95% CI)	*P* value	HR(95% CI)	*P* value	HR(95% CI)	*P* value
**Dietary Spermidine**	Q1	1.00 (ref.)	-	1.00 (ref.)	-	1.00 (ref.)	-
	Q2	0.82(0.66, 1.03)	0.096	1.04(0.72, 1.50)	0.828	0.68(0.42, 1.10)	0.115
	Q3	0.83(0.66, 1.05)	0.121	0.92(0.63, 1.33)	0.647	0.58(0.35, 0.97)	0.036
	Q4	0.71(0.56, 0.91)	0.005	0.74(0.50, 1.09)	0.132	0.55(0.33, 0.91)	0.021
	Q5	0.95(0.74, 1.23)	0.704	1.06(0.70, 1.61)	0.770	0.59(0.32, 1.06)	0.078
**Dietary Spermine**	Q1	1.00 (ref.)	-	1.00 (ref.)	-	1.00 (ref.)	-
	Q2	0.79(0.63, 0.99)	0.040	0.58(0.41, 0.82)	0.002	0.85(0.51, 1.41)	0.527
	Q3	0.77(0.61, 0.97)	0.024	0.52(0.36, 0.74)	<0.001	0.82(0.50, 1.37)	0.455
	Q4	0.86(0.69, 1.08)	0.205	0.78(0.56, 1.08)	0.129	0.66(0.39, 1.13)	0.133
	Q5	0.91(0.71, 1.19)	0.503	0.70(0.47, 1.04)	0.076	0.62(0.33, 1.17)	0.138
**Dietary Putrescine**	Q1	1.00 (ref.)	-	1.00 (ref.)	-	1.00 (ref.)	-
	Q2	0.91(0.72, 1.15)	0.436	0.67(0.48, 0.95)	0.024	0.09(0.51, 1.57)	0.703
	Q3	1.05(0.83, 1.33)	0.694	0.62(0.44, 0.89)	0.009	1.04(0.6, 1.81)	0.880
	Q4	0.96(0.76, 1.22)	0.753	0.87(0.63, 1.22)	0.425	0.63(0.34, 1.15)	0.131
	Q5	0.92(0.70, 1.22)	0.567	0.60(0.39, 0.91)	0.016	0.76(0.39, 1.47)	0.409

Abbreviations: HR, hazard ratio; CI, confidence interval; -: Not available; AD, Alzheimer's disease; VD, vascular dementia; Ref , reference.

Analyses were adjusted for age, sex, socioeconomic status (least deprived/medium/most deprived), education (high/other), smoke status (never/previous/current), alcohol intake (<1 time/week, 1–2 times/week, 3–4times/week, daily or almost daily), energy, sleep duration (<7 hours/7–8 hours/>8 hours), physical activity levels (moderate/low/high), polygenic risk score, APOE ε4, hypertension (no/yes), hypercholesteremia (no/yes), diabetes (no/yes) and first 10 principal components of ancestry.

Table S9. Sensitivity analyses adjusting for potential effect mediators (Depression).

Polyamines		All-cause dementia		AD		VD	
		HR(95% CI)	*P* value	HR(95% CI)	*P* value	HR(95% CI)	*P* value
**Dietary Spermidine**	Q1	1.00 (ref.)	-	1.00 (ref.)	-	1.00 (ref.)	-
	Q2	0.78(0.64, 0.93)	0.008	0.86(0.65, 1.14)	0.286	0.70(0.46, 1.05)	0.084
	Q3	0.79(0.65, 0.95)	0.013	0.76(0.56, 1.02)	0.063	0.60(0.39, 0.93)	0.021
	Q4	0.68(0.56, 0.83)	<0.001	0.62(0.45, 0.85)	0.003	0.56(0.36, 0.87)	0.010
	Q5	0.87(0.72, 1.05)	0.157	0.84(0.62, 1.12)	0.214	0.62(0.41, 0.95)	0.030
**Dietary Spermine**	Q1	1.00 (ref.)	-	1.00 (ref.)	-	1.00 (ref.)	-
	Q2	0.76(0.63, 0.91)	0.004	0.60(0.45, 0.80)	0.001	0.85(0.56, 1.28)	0.433
	Q3	0.74(0.61, 0.89)	0.002	0.53(0.39, 0.72)	<0.001	0.82(0.54, 1.24)	0.356
	Q4	0.82(0.68, 1.00)	0.045	0.80(0.61, 1.05)	0.105	0.66(0.42, 1.04)	0.070
	Q5	0.83(0.68, 1.00)	0.049	0.65(0.49, 0.88)	0.004	0.64(0.41, 1.02)	0.057
**Dietary Putrescine**	Q1	1.00 (ref.)	-	1.00 (ref.)	-	1.00 (ref.)	-
	Q2	0.77(0.64, 0.93)	0.008	0.68(0.51, 0.91)	0.010	0.63(0.42, 0.96)	0.033
	Q3	0.89(0.74, 1.07)	0.228	0.64(0.47, 0.86)	0.003	0.76(0.51, 1.14)	0.190
	Q4	0.82(0.68, 0.99)	0.044	0.89(0.68, 1.18)	0.415	0.47(0.29, 0.75)	0.002
	Q5	0.85(0.70, 1.03)	0.088	0.70(0.52, 0.95)	0.019	0.74(0.49, 1.12)	0.157

Abbreviations: HR, hazard ratio; CI, confidence interval; -: Not available; AD, Alzheimer's disease; VD, vascular dementia; Ref , reference.

Analyses were adjusted for age, sex, socioeconomic status (least deprived/medium/most deprived), education (high/other), smoke status (never/previous/current), alcohol intake (<1 time/week, 1–2 times/week, 3–4times/week, daily or almost daily), energy, sleep duration (<7 hours/7–8 hours/>8 hours), physical activity levels (moderate/low/high), polygenic risk score, APOE ε4, hypertension (no/yes), hypercholesteremia (no/yes), diabetes (no/yes) and first 10 principal components of ancestry, depression.

Table S10. Sensitivity analyses adjusting for potential effect mediators (BMI).

Polyamines		All-cause dementia		AD		VD	
		HR(95% CI)	*P* value	HR(95% CI)	*P* value	HR(95% CI)	*P* value
**Dietary Spermidine**	Q1	1.00 (ref.)	-	1.00 (ref.)	-	1.00 (ref.)	-
	Q2	0.77(0.64, 0.93)	0.007	0.86(0.64, 1.14)	0.280	0.69(0.46, 1.04)	0.080
	Q3	0.79(0.65, 0.95)	0.012	0.75(0.56, 1.01)	0.060	0.60(0.39, 0.93)	0.022
	Q4	0.68(0.56, 0.82)	<0.001	0.62(0.45, 0.84)	0.002	0.57(0.37, 0.88)	0.012
	Q5	0.87(0.72, 1.05)	0.134	0.83(0.62, 1.11)	0.214	0.63(0.41, 0.96)	0.034
**Dietary Spermine**	Q1	1.00 (ref.)	-	1.00 (ref.)	-	1.00 (ref.)	-
	Q2	0.76(0.63, 0.91)	0.004	0.06(0.45, 0.80)	0.001	0.85(0.56, 1.28)	0.427
	Q3	0.73(0.61, 0.89)	0.002	0.53(0.39, 0.72)	<0.001	0.82(0.54, 1.24)	0.342
	Q4	0.82(0.68, 1.00)	0.045	0.8(0.61, 1.05)	0.112	0.66(0.42, 1.03)	0.070
	Q5	0.83(0.68, 1.00)	0.052	0.66(0.49, 0.88)	0.005	0.64(0.41, 1.02)	0.059
**Dietary Putrescine**	Q1	1.00 (ref.)	-	1.00 (ref.)	-	1.00 (ref.)	-
	Q2	0.77(0.64, 0.93)	0.007	0.68(0.51, 0.91)	0.01	0.63(0.42, 0.96)	0.033
	Q3	0.89(0.74, 1.07)	0.216	0.63(0.47, 0.86)	0.003	0.76(0.51, 1.15)	0.194
	Q4	0.82(0.68, 0.99)	0.039	0.89(0.67, 1.17)	0.398	0.47(0.29, 0.75)	0.002
	Q5	0.84(0.70, 1.02)	0.077	0.70(0.52, 0.94)	0.019	0.74(0.49, 1.12)	0.152

Abbreviations: HR, hazard ratio; CI, confidence interval; -: Not available; AD, Alzheimer’s disease; VD, vascular dementia; Ref , reference.

Analyses were adjusted for age, sex, socioeconomic status (least deprived/medium/most deprived), education (high/other), smoke status (never/previous/current), alcohol intake (<1 time/week, 1–2 times/week, 3–4times/week, daily or almost daily), energy, sleep duration (<7 hours/7–8 hours/>8 hours), physical activity levels (moderate/low/high), polygenic risk score, APOE ε4, hypertension (no/yes), hypercholesteremia (no/yes), diabetes (no/yes) and first 10 principal components of ancestry, bmi (normal/overweight/obese).

Table S11. Sensitivity analyses adjusting for potential effect mediators (Stroke).

**Polyamines**		**All-cause dementia**		**AD**		**VD**	
		**HR(95% CI)**	***P* value**	**HR(95% CI)**	***P* value**	**HR(95% CI)**	***P* value**
**Dietary Spermidine**	Q1	1.00 (ref.)	-	1.00 (ref.)	-	1.00 (ref.)	-
	Q2	0.80(0.66, 0.97)	0.020	0.85(0.64, 1.13)	0.258	0.70(0.46, 1.05)	0.084
	Q3	0.80(0.66, 0.97)	0.023	0.72(0.54, 0.97)	0.032	0.53(0.34, 0.83)	0.006
	Q4	0.70(0.57, 0.85)	<0.001	0.58(0.42, 0.79)	0.001	0.57(0.36, 0.89)	0.014
	Q5	0.91(0.75, 1.09)	0.302	0.82(0.61, 1.11)	0.195	0.65(0.42, 1.00)	0.051
**Dietary Spermine**	Q1	1.00 (ref.)	-	1.00 (ref.)	-	1.00 (ref.)	-
	Q2	0.76(0.63, 0.92)	0.005	0.59(0.44, 0.79)	<0.001	0.91(0.60, 1.39)	0.686
	Q3	0.74(0.61, 0.90)	0.002	0.56(0.41, 0.76)	<0.001	0.77(0.50, 1.18)	0.229
	Q4	0.85(0.70, 1.03)	0.094	0.82(0.62, 1.09)	0.169	0.68(0.43, 1.07)	0.097
	Q5	0.82(0.68, 1.00)	0.047	0.65(0.49, 0.87)	0.004	0.69(0.43, 1.09)	0.115
**Dietary Putrescine**	Q1	1.00 (ref.)	-	1.00 (ref.)	-	1.00 (ref.)	-
	Q2	0.80(0.67, 0.97)	0.025	0.69(0.52, 0.93)	0.014	0.68(0.45, 1.05)	0.084
	Q3	0.90(0.74, 1.08)	0.244	0.63(0.46, 0.85)	0.003	0.68(0.45, 1.03)	0.065
	Q4	0.81(0.67, 0.98)	0.034	0.83(0.63, 1.1)	0.201	0.47(0.29, 0.76)	0.002
	Q5	0.80(0.66, 0.97)	0.024	0.67(0.5, 0.9)	0.009	0.65(0.42, 0.99)	0.040

Abbreviations: HR, hazard ratio; CI, confidence interval; -: Not available; AD, Alzheimer's disease; VD, vascular dementia Ref , reference.

Analyses were adjusted for age, sex, socioeconomic status (least deprived/medium/most deprived), education (high/other), smoke status (never/previous/current), alcohol intake (<1 time/week, 1–2 times/week, 3–4times/week, daily or almost daily), energy, sleep duration (<7 hours/7–8 hours/>8 hours), physical activity levels (moderate/low/high), polygenic risk score, APOE ε4, hypertension (no/yes), hypercholesteremia (no/yes), diabetes (no/yes) and first 10 principal components of ancestry, stroke.
